# Genomic epidemiology of Mycobacterium tuberculosis in Malawi: using global phylogeography to understand the impact of geographically focused interventions

**DOI:** 10.1099/mgen.0.001674

**Published:** 2026-04-08

**Authors:** Alexander B. Beams, Yexuan Song, Jennifer McNichol, Bradley R. Jones, Benjamin Sobkowiak, Peter MacPherson, Marriott Nliwasa, Victor Ndhlovu, Mphatso D. Phiri, Ted Cohen, Caroline Colijn

**Affiliations:** 1 Department of Mathematics, Simon Fraser University, Burnaby, Canada; 2 Infection, Immunity and Inflammation Dept, University College London, London, UK; 3 School of Health & Wellbeing, University of Glasgow, Glasgow, UK; 4 Malawi Liverpool Wellcome Programme, Blantyre, Malawi; 5 Clinical Research Department, London School of Hygiene & Tropical Medicine, London, UK; 6 Division of Epidemiology and Biostatistics, School of Public Health, Faculty of Health Sciences, University of the Witwatersrand, Johannesburg, South Africa; 7 Helse Nord Clinical Research and Training Initiative, Kamuzu University of Health Sciences, Blantyre, Malawi; 8 Kamuzu University of Health Sciences, Blantyre, Malawi; 9 Liverpool School of Tropical Medicine, Liverpool, UK; 10 Department of Epidemiology of Microbial Diseases, Yale School of Public Health, Yale University, New Haven, CT, USA

**Keywords:** ancestral state inference, genomic epidemiology, phylogenetics, *Mycobacterium tuberculosis*, tuberculosis, whole-genome sequencing

## Abstract

Genomic surveillance of pathogens important to public health, such as *Mycobacterium tuberculosis* (Mtb), offers the opportunity to characterize the geographic movements of pathogens on a range of spatial and temporal scales and to explore the consequences of these inferred movements on the impacts of public health interventions. Pathogen movements can affect the impact of interventions that are geographically focused, with interventions in high-transmission areas, potentially leading to indirect benefits in other locations due to the prevention of transmission. We supplemented a large genomic surveillance dataset from Blantyre, Malawi (518 Lineage 4 sequences and 103 Lineage 1 sequences) with publicly available sequences collected across the world from 2015 to 2019 (910 Lineage 4 sequences and 445 Lineage 1 sequences) to reconstruct global and regional movements of Mtb in order to clarify the extent of importation into Blantyre. Standard phylogeographic methods are unsuitable for this task because they do not account for sampling heterogeneity across locations, so we build on a new method called sampling-aware ancestral state inference, incorporating wide disparities in the sampling fractions between different regions across the world, and also the fact that sampling only occurred from 2015 to 2019. Reconstructed phylogenetic trees contain strong signals of spatial localization of individual clades, with very limited numbers of introductions to, or exports from, Blantyre, and considerable movements within the city itself. Inferring which zone of the Blantyre nodes of the tree was in allows us to perform simple simulations of geographically focused interventions such as active case finding (ACF). We find that zone-focused ACF in Blantyre is likely to have modest impacts, with a focus on Zones 2 and 4 likely to have the most impact. As genomic surveillance becomes more commonplace, analyses such as this may be useful for public health practitioners developing interventions to reduce local TB transmission and incidence.

**Impact Statement**

Infections caused by *Mycobacterium tuberculosis* (Mtb) are responsible for millions of deaths across the world annually. Infection progresses to a symptomatic and infectious stage after a long period of latency that can persist for years. From the point of view of public health, the possibility that an individual may travel a long distance from their source of infection in the intervening time obscures epidemiological links and may undermine geographically targeted interventions seeking to limit transmission. Genomic data can help resolve the spatial migration patterns of Mtb lineages using phylogeographic analysis. Phylogeography aims to reconstruct the likely locations of ancestors represented in phylogenies using information about the locations of sampled infections. We analyse a dataset consisting of sequences from infections in Blantyre, Malawi, collected between 2015 and 2019, and supplemented with sequences across the world during the same period of time. We use a method called sampling-aware ancestral state inference to account for different intensities of sampling in Malawi vs. the rest of the world. Our estimates identify a few lineage movements into Malawi from the rest of the world. However, simulations show that interventions designed to prevent onward transmission within particular neighbourhoods of the city of Blantyre have modest effectiveness. This shows that transmission networks operate at spatial scales intermediate in size between city and country. The majority of new infections likely arise from transmission events within the country, emphasizing the importance of national efforts to reduce infections.

## Data Summary

Codes and a version of the data with Blantyre zone information redacted to protect patient privacy are available at the GitHub repository located at https://github.com/MAGPIE-SFU/tb-malawi-phylogeo-public. By virtue of redaction of granular location-time combinations that would endanger patient privacy, interested researchers will not be able to replicate the analysis herein in full, but will be able to quantify lineage movements between large geographic regions. The Blantyre sequences are publicly available and maintained by the National Center for Biotechnology Information under BioProject number PRJNA1221228(https://www.ncbi.nlm.nih.gov/bioproject/?term=PRJNA1221228). The international sequences in this study were publicly available at the European Nucleotide Archive (https://www.ebi.ac.uk/ena/browser/home). Accession numbers for all sequences are included as supplementary CSV files (separated by lineage). The time of collection of sequences in the Blantyre dataset has been labelled with the Wednesday of the week in which the sample was collected. A copy of this repository has also been uploaded to FigShare 10.6084/m9.figshare.31372795 [1].

## Introduction

Public health initiatives have produced impressive reductions in tuberculosis (TB) incidence across Africa, but declines in incidence appear to be slowing [2,3]. Nations with more limited resources experience unique challenges in eliminating TB, including poor access to antimicrobials and underfunded public health programmes [3]. In places like Blantyre, Malawi, TB prevalence has decreased over the past decade through rapid population scale-up of antiretroviral therapy for HIV and a well-functioning public health programme, but there is evidence to suggest that people with undiagnosed TB are becoming increasingly difficult to detect, possibly because they are increasingly concentrated in areas or risk groups that public health programmes find challenging to access [4]. In some cases, there is evidence that TB transmission is geographically clustered, and analyses of mathematical models indicate that reducing transmission in these hotspots can be very cost-effective for reducing overall prevalence [5]. Owing to its long latent period, however, the spread of TB is intrinsically linked with human movement, and it is possible that patterns of population movement within cities and more widely could undermine efforts to develop local, targeted interventions [6].

In general, disease prevalence becomes patchy in space as prevalence declines [7], and identifying remaining transmission hotspots becomes increasingly difficult [8]. Active case finding (ACF) is one component of the End TB strategy that comprises a suite of public health interventions, including enhanced screening and community outreach, to identify individuals with undiagnosed TB. However, ACF is expensive and requires strong relationships between public health institutions and the people they serve [9]. Temporary ACF initiatives usually result in increases in case detection and, if sufficiently intense, reductions in prevalence in the short term; but achieving End TB targets (as well as TB elimination) will require continuous improvements in ACF effectiveness over time (as measured in terms of undiagnosed cases found and treated) [10]. However, determining ACF effectiveness in practice has proved challenging, and impacts on transmission are unclear. Cluster-randomized trials show that screening independent of symptoms can produce elevated notification rates but symptoms-driven screening may not (c.f. the ACT3 trial in Vietnam and the ZAMSTAR trial in Zambia and South Africa) and do not identify clear changes in TB incidence in children after interventions, suggesting they do not substantially reduce transmission [11]. A cluster-randomized trial was also carried out in 2019–2020 in Blantyre to compare ACF with passive case detection systems already in place, but low TB prevalence and the Coronavirus disease 2019 (COVID-19) pandemic made estimation difficult [12]. In other settings, screening programmes for TB sometimes identify and treat cases earlier than passive surveillance, leading to better patient outcomes and reduced overall costs of treatment, but the evidence for effectiveness is mixed overall [13]. Where it is effective, ACF appears to improve both the likelihood of successful treatment (OR=1.4 relative to passive surveillance [14]) and to reduce overall costs of treatment (probably by identifying infections earlier [15]). In general, the effectiveness of ACF depends on local prevalence, the intensity and coverage of public health responses and metrics used to gauge success [9].

Genomic surveillance and a range of research studies on *Mycobacterium tuberculosis* (Mtb), the causative agent of TB, have produced large amounts of publicly available sequence data, in particular through the European Nucleotide Archive (ENA) [16], which can improve the understanding of patterns of Mtb’s transmission and hence the impacts of local interventions aiming to reduce transmission. For Mtb, which has a variable latent period that can last for several years, elevated notification rates are not always reliable indicators of heightened transmission because underlying spatial distributions of risk factors for progression to active disease and differential access to diagnosis and treatment can contribute to elevated case counts [17]. Genomic data can be useful for distinguishing these effects [18]. Likewise, comparable timescales for human movements and disease transmission/progression imply that a substantial portion of TB notifications in some jurisdictions may be imported from elsewhere (as appears to be the case, for example, in Ecuador [19]). In Malawi, the extent of importation of Mtb is unclear and may differ in rural versus urban settings. For example, genomic analysis of infections in rural Malawi suggests that Mtb Lineage 4 infections are more likely to occur in local transmission events than other lineages [20], but analysis of data from the city of Blantyre is consistent with Lineage 4 being imported more frequently than other lineages [21]. The finer spatiotemporal dynamics within the city of Blantyre are better understood: genomic analysis of data from the city identifies some transmission networks that are geographically localized within the city but also reveals substantial amounts of transmission occurring across different neighbourhoods, making it unclear whether interventions targeted to particular neighbourhoods inside the city are likely to be effective [22]. The extent to which Mtb transmission primarily remains within Malawi (once established there) is unclear, but analysing global sequence data from ENA [16] may be useful for determining this.

The effectiveness of local interventions to reduce TB incidence in Blantyre depends in part on the amount of interconnection with larger regions. Phylogeographic analysis of genomic data can be useful for quantifying these interconnections, but standard phylogeography methods require restrictive assumptions. Typical approaches use mathematical models to perform ancestral character estimation, which infers probable locations of ancestral infections. However, these models assume that infections in any location have the same probability of being observed, which can lead to large biases if sampling is heterogeneous across locations and over time [23,24]. This is a major drawback for studying Mtb importations into Blantyre (and most other locations) because data collection is highly heterogeneous across the world. However, recent developments in phylogeographic analysis extend existing models to carry out sampling-aware ancestral state inference (SAASI) [24]. We adopt this new approach to set the transmission of Mtb in Blantyre within the larger global context. In this paper, we analyse data from Mtb Lineage 1 and 4 infections in Blantyre, Malawi, collected from 2015 to 2019 (described in [22]). We supplement this Blantyre-centred data with sequences collected across the world available from the ENA in a manner approximately representative of the number of notifications by lineage. We carry out SAASI to infer probable locations of ancestral Mtb infections in the recent past in order to estimate the relative amount of transmission within the city of Blantyre vs. the extent of importation into the city from other locations and then pair our analysis with a simulation to assess how well geographically focused interventions like ACF might impact transmission in the city.

## Methods

### Blantyre data

**Fig. 1.**
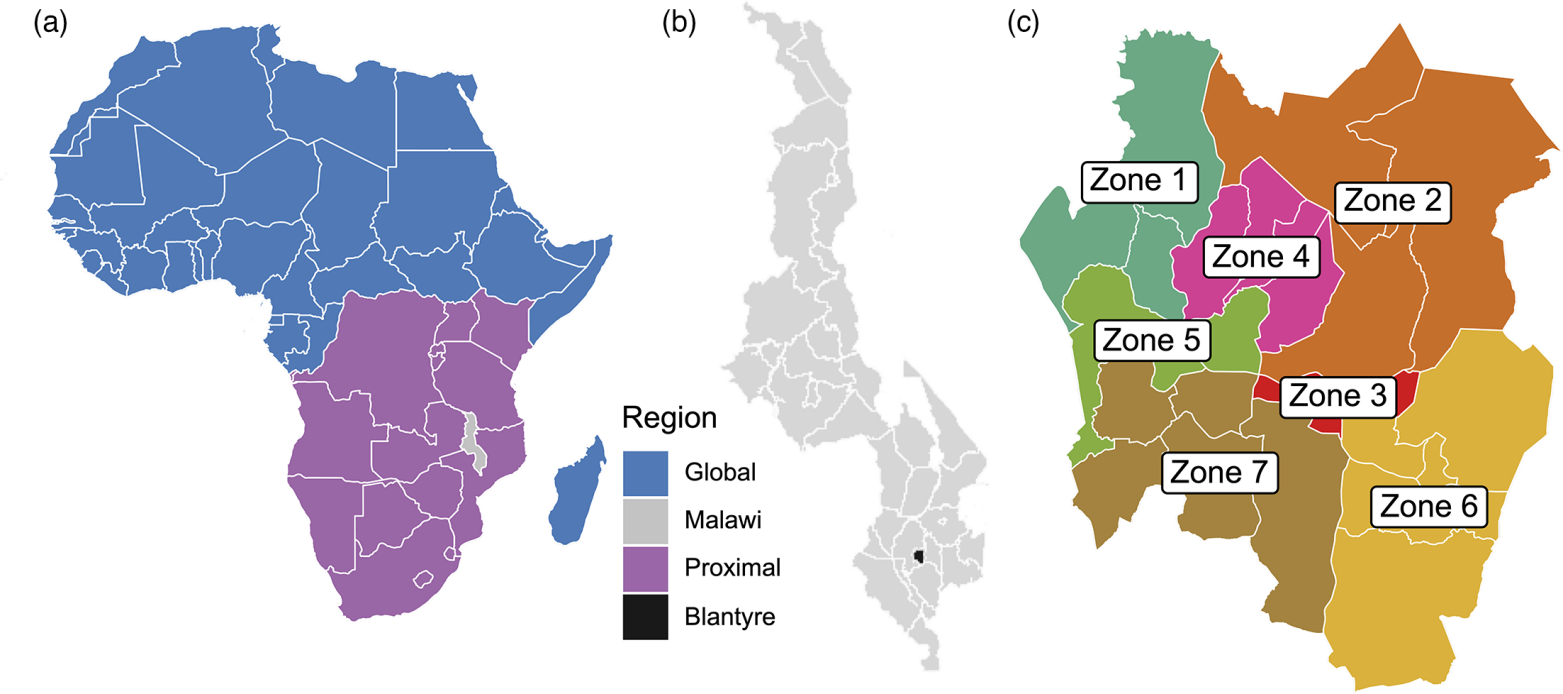
Map of geographic regions in Africa included in the analysis. Global sequences in Africa belong to the same category as sequences sampled from North and South America, Europe and Asia. (**a**) Proximal countries (purple) are those that have the most land-based travel to and from Malawi; all other countries in Africa are considered part of the Global sample group (blue). (**b**) Within Malawi, the data are collected from a single city, Blantyre, (**c**) which is further stratified into different zones comprising multiple wards. Sequences in the analysis consist of Lineage 4 [518 from Blantyre, 232 from Proximal (*b*=1) or 464 from Proximal (*b*=2), 346 from Global and 100 from countries in the global region with low sequence availability (Global-LSA)] and Lineage 1 [103 from Blantyre, 31 from Proximal (*b*=1) or 41 from Proximal (*b*=2), 93 from Global and 311 from Global-LSA].

Our data from Blantyre, analysed and described previously [22], were collected through enhanced surveillance of people notified with TB at health centres and hospitals throughout Blantyre between 1 January 2015 and the end of 2019. There are 518 *Mycobacterium tuberculosis* complex Lineage 4 isolates, 103 Lineage 1 isolates, 28 Lineage 2 isolates and 62 Lineage 3 isolates. Here, we focus on the most prevalent lineages 1 and 4 isolates. All sequences have a zone identifier indicating location (zone) within the city of Blantyre (Table S1, available in the online Supplementary Material, and [22]). Blantyre has seven zones (defined in partnership with the Malawi National TB and Leprosy Elimination Programme) that each comprise multiple wards (Fig. 1). These data were previously analysed using methods that reconstruct transmission networks to determine spatial structure in Mtb transmission, and these identified substantial between-zone transmission within Blantyre. A small number of individuals in the Blantyre data have unassigned zone location, so we assign them to other locations using other available information, assign them to zones randomly or drop them from the analysis to see how results change (see Material S1). The majority of Lineage 4 sequences in the Blantyre data belong to sublineage 4.3, although most sublineages are present (except for sublineage 4.7).

### International data

We obtain international data publicly available through the ENA [16] from the time period 2015–2019 (Table S2). The data that we download from ENA consist only of those sublineages that also occur in the Blantyre data. As our aim is phylogeographic reconstruction and characterization of Mtb movements, it is particularly important to include sequences from nearby countries with high volumes of travel to and from Malawi. In 2019, most international visitors arrived from Mozambique, Zimbabwe, Tanzania, Zambia, South Africa and Kenya, followed by the USA and other non-African countries [25]. The six African countries either border Malawi or have a land border with a country that borders Malawi, and large numbers of migrant workers travel by land between the countries. However, of these six countries, sequences are only available from South Africa, Kenya and Tanzania. For our analyses, we pool these countries with sequences from Botswana, the Democratic Republic of the Congo, Uganda and Rwanda into a region we refer to as ‘Proximal’ (see Fig. 1). There is a high volume of land-based travel between Malawi and other nearby countries in the Proximal region due to migration related to employment and food insecurity [26], as well as informal cross-border exchange that is often mediated by individual women traders [27,28]. Therefore, we expect a large number of lineage movements between Blantyre and the proximal region. Hence, we analyse two datasets, one of which has enriched sampling from the proximal region (‘*b*=2’) to allow for stronger representation of the nearest regions to Malawi, compared to a baseline analysis (‘*b*=1’) in which data from the proximal region are not enriched.

We compute the total mean annual number of Mtb notifications for each region or country in the 2015–2019 time period [29]. We denote the mean annual notifications $N_{j}$, where N is notifications and j denotes region (the proximal region is treated as one location). For Mtb Lineages 1 and 4, we calculate notifications by lineage over the relevant time period using the fraction of ENA sequences from a country in the given lineage to scale the country’s notifications. For example, we set $N_{j}^{4}=f_{4}N_{j}$, where $f_{j}^{4}$ is the fraction of the country j’s sequences that are in Lineage 4. For proximal countries with no available sequences, we use Blantyre’s fraction of sequences to determine $f_{j}^{4}$ and $f_{j}^{1}$. We omit non-proximal countries for which no sequences are available.

It would be optimal to select sequences from countries approximately in proportion to their contribution to the global Mtb burden (by lineage) as estimated by the World Health Organization (WHO) [29], but this is not always possible because of limited capacity to test and sequence Mtb isolates. Our selection process is therefore as follows. Suppose that we wish to complement our Blantyre data with $N_{tot}$ additional sequences. For proportional sampling, the number of (Lineage 4, for example) sequences from the country j that we would wish to include in our analysis is


$$W_{j}=N_{tot}\frac{N_{j}^{4}}{\sum_{j}N_{j}^{4}}.$$

For some countries, however, there are fewer than $W_{j}$ available sequences from our time period. For these countries, we take the lower sampling fraction into account in our phylogeographic analysis. We group such countries into a ‘low sequence availability’ (Global-LSA) category and retain all of their available sequences. For the analysis with enriched representation from the proximal region, we set $W_{\text{proximal}}=bN_{tot}\frac{N_{\text{proximal}}^{4}}{\sum_{j}N_{j}^{4}}$ with b=2. We show the analysis with b=1 in the Material S1.

This process results in four regions: Global, Global-LSA, Proximal and Blantyre (further divided into seven zones). In the Global, Global-LSA and Proximal regions, we have approximate sampling fractions, namely the ratio of the number of sequences we have in a given lineage to the total estimated WHO notifications for that lineage. In Blantyre, we use the proportion of notifications sequenced between 1 January 2015 and the end of 2019. We can account for these differences in sampling in our phylogeographic analysis.

### Variant calling and phylogenetics

We downloaded raw sequencing data for international Mtb isolates with country and full or partial date metadata from the National Center for Biotechnology Information using the SRA toolkit (Table S2). We use these to supplement sequences collected in Blantyre from 2015 to 2019, described previously [22]. Sequencing data are aligned to the Mtb H37Rv reference genome (NC_000962.3) using the BWA ‘mem’ algorithm [30], and binary alignment files are produced using SAMtools [31]. Mapping statistics are calculated using the SAMtools ‘flagstat’, and the number of Mtb strains present in each sample is predicted using MixInfect2 [32]. Samples with evidence of multiple strains (mixed infection) and with §amp;lt;80% of reads mapping to the reference sequence are removed. Of the sequences we select for analysis from ENA, ~5% have evidence of mixed infection and are removed. Where $W_{j}$ is lower than the number of lineage-specific isolates available from a country, a random subset of $W_{j}$ isolates from the country is chosen.

Variant calling is performed with GATK [33] to identify high-confidence SNPs with read depth ≥5 and ≥80% of reads supporting the consensus allele to produce a multi-sequence alignment of SNPs. These alignments are used to create maximum-likelihood (ML) phylogenies for each dataset using IQ-TREE 2 [34], with 1,000 bootstrap resamples and using the ‘-m TEST’ option to find the optimal nucleotide substitution model. Finally, dated phylogenies are built from the ML trees for each dataset with BactDating [35], scaled using the full or partial collection dates at the tips. The model is run for each tree for $10^{6}$ MCMC iterations using a ‘mixgamma’ clock model to estimate the standard deviation of the per-branch substitution rates (σ; see [35]) while keeping the mean substitution rate fixed at 0.5 SNPs/genome/year [36]. We compare branch lengths in the resulting timed phylogenies with those obtained under a fixed mean substitution rate of 0.3 SNPs/genome/year [37].

### Phylogeographic analysis

To infer the geographic locations of infections ancestral to our sample, we use SAASI [24]. SAASI generalizes Markovian models of discrete trait evolution to accommodate heterogeneous sampling rates across trait values. It uses a similar likelihood function as state-dependent speciation and extinction models that allow diversification rates to vary with traits [38] and is tractable for large trees (§amp;gt;500 taxa). SAASI requires specification of speciation, extinction and sampling rates, as well as a transition rate matrix to capture Markovian transition probabilities among discrete traits.

We consider sampling rates $\psi_{i}(t)$ that vary in a piecewise manner over two discrete time intervals in our analysis. SAASI allows for a set of n+1 time points $\left(t_{0}=0,t_{1},t_{2},\cdots ,t_{n-1},t_{n}={\text{T}}_{\text{MRCA}}\right)$ measured from the present day to the root, each with a sampling rate $\psi_{i}(t)$ for state i at time t:


$$\psi_{i}(t):=\psi_{i,k}\;\;\text{for}\;t\in [t_{k-1},t_{k}),$$

where $\psi_{i,k}$ is the constant sampling rate for the state i during the time interval $\left[t_{k-1},t_{k}\right)$.

Our data begin in 2015, so we set sampling rates equal to zero during the period before 2015. We set three time points ($t_{0}=0,t_{1}=5,t_{2}={\text{T}}_{\text{MRCA}}$) in our phylogeographic analysis. Here, 0 represents the final sampling time (end 2019), $t_{1}=5$ corresponds to the beginning of 2015 and $t_{2}$ corresponds to the earliest time in a tree (the root time). For $t\in [t_{0},t_{1})$, we use the estimated sampling rate described in the ‘Sampling rates for SAASI’ section, and we set $\psi_{i}(t)=0$ for all states i for $t\in [t_{1},t_{2})$. See Table 1 for values used in the analysis.

**Table 1.** Estimated speciation (*λ*), extinction (*μ*) and sampling rates (*ψ*) for birth-death sampling models fit to the phylogenies All rates are reported in units of year^−1^.

	$\hat{\lambda }$ (std. error) per year		$\hat{\mu }$ (std. error) per year	$\hat{\psi }$ (std. error)	
Proximal	Lineage 4	Lineage 1	Both lineages	Lineage 4	Lineage 1
b=1	$2\times 10^{-3}$ $\left(8\times 10^{-4}\right)$	$4\times 10^{-3}$ $\left(4\times 10^{-4}\right)$	$10^{-3}$ (L4: $8\times 10^{-5}$, L1: $9\times 10^{-5}$)	$9\times 10^{-4}$ $\left(8\times 10^{-5}\right)$	$5\times 10^{-4}$ $\left(9.6\times 10^{-5}\right)$
b=2	$3\times 10^{-3}$ $\left(6\times 10^{-5}\right)$	$4\times 10^{-3}$ $\left(8\times 10^{-5}\right)$	$10^{-3}$ (L4: $8\times 10^{-5}$, L1: $2\times 10^{-8}$)	$2\times 10^{-3}$ $\left(2\times 10^{-8}\right)$	$5\times 10^{-4}$ $\left(9.6\times 10^{-5}\right)$

### Diversification rates for SAASI

SAASI performs ancestral state inference using a birth-death-sampling model based on the binary state-dependent speciation and extinction (BiSSE) family of models [39] with extension to incompletely observed phylogenies [38,40]. In principle, each state (here, geographic regions) may have its own diversification rate, death rate and sampling rate. The BiSSE family of models takes into account the likelihood of observing a tree descending from a node conditional on that node residing in a particular state. For example, if a node is in a state with a low sampling rate, a descending tree with long branches and few sampled tips is more likely than it would be if the node were in a state with a high sampling rate. Similarly, SAASI accounts for the likelihood that a node is in a particular state given the tree from which it descends. For a full description of SAASI, see [24]. SAASI requires an overall branching (or diversification) rate (λ) and an overall extinction (or, in infectious disease applications, a recovery) rate (μ), as well as sampling rates ($\psi_{i}$) for each state in each time interval. We estimate λ and μ using an ML method proposed by [41], implemented in the SAASI package [24]. We denote the estimates $\hat{\lambda }$ and $\hat{\mu }$. This method also estimates an overall sampling rate $\hat{\psi }$ (which is not state-dependent).

We set λ and μ independent of the states. The state-dependent sampling rate estimation is described in the ‘Sampling rates for SAASI’ section. For Lineage 4 with b=2, the estimated sampling rates for the Blantyre zones are $9\times 10^{-4}$, which is of the same magnitude as $\hat{\lambda },\hat{\mu }$ and $\hat{\psi }$. Because the vast majority of Mtb is expected to occur in the Global region, we consider the consequences of allowing λ to be an order of magnitude larger in the Global state relative to the rest (see Table S3).

### Sampling rates for SAASI

Notified Mtb infections from the different regions in our data (zones within Blantyre, countries in Africa Proximal to Malawi, Global and Global – low sequence availability) have different probabilities of sequencing and inclusion in our data. SAASI requires specification of a sampling rate ($\psi_{i}$) for each location, though we allow $\psi_{i}$ to vary over time in a piecewise constant manner. To obtain sampling rates with units per year, we convert available fractions of notified infections sequenced ($f_{i}$) to sampling rates (here, this means to be sequenced and in our dataset) per year ($\psi_{i}$). Specifically, we relate sampling fraction, $f_{i}$, to yearly rate of sampling, $\psi_{i}$, through the relationship $f_{i}=1-e^{-5\psi_{i}}$ (sequences were collected over a 5-year period from 2015 through the end of 2019). For both Lineages 1 and 4, this produces yearly sampling rates smaller than $10^{-2}$ for all regions, but with notified Mtb infections in Blantyre sampled at a rate nearly 100 times higher than the other regions (Table 2).

**Table 2.** Sequencing fractions and calculating sampling rates for sequences in the dataset The sequencing rates per year (‘sequencing rate’, *$\psi_{i}$*) are calculated from the fractions of notifications sequenced (*$f_{i}$*) as*$f_{i}=1-e^{-5\psi_{i}}$*.

	Fraction of notifications sequenced		Sequencing rate (year^−1^)		Number of sequence	
Region	Lineage 4	Lineage 1	Lineage 4	Lineage 1	Lineage 4	Lineage 1
Blantyre	$4.46\times 10^{-2}$	$4.46\times 10^{-2}$	$9.13\times 10^{-3}$/year	$9.13\times 10^{-3}$/year	518	103
Proximal b=1	$4.40\times 10^{-4}$	$7.71\times 10^{-4}$	$8.80\times 10^{-5}$/year	$1.54\times 10^{-4}$/year	232	31
Proximal b=2	$8.80\times 10^{-4}$	$1.02\times 10^{-3}$	$1.76\times 10^{-4}$/year	$2.04\times 10^{-4}$/year	464	41
Global	$4.18\times 10^{-4}$	$6.92\times 10^{-4}$	$8.37\times 10^{-5}$/year	$1.38\times 10^{-4}$/year	346	93
Global-LSA	$2.56\times 10^{-4}$	$3.63\times 10^{-4}$	$5.13\times 10^{-5}$/year	$7.25\times 10^{-5}$/year	100	311

We also calculate sampling rates from sampling fractions in two other ways. In both cases, we assume the existence of an overall sampling fraction proportional to the estimated diversification rate (λ≈0.01). We calculate sampling rates either as $\psi_{i}=x\lambda f_{i}$ or as $\psi_{i}=x\lambda f_{i}/\sum_{j}f_{j}$. The magnitudes of these rates are smaller than those calculated in Table 2, but the relative sizes of the rates are similar to those calculated using the exponential relationship between sampling fraction and rate. The sampling rates from these estimates for Blantyre are higher than those estimated by the MLE method in the SAASI package, but in birth-death-sampling models, sampling is a form of removal. If the sampling rate is set too high, the net growth in the birth-death process is insufficient to match the number of lineages. Therefore, for consistency with the birth-death-sampling model in the MLE, in our analyses, we adjust all estimated sequencing rates downwards by a constant factor. We use these adjusted rates, reported in Table 2. We also examine the estimates $\hat{\lambda }$ and $\hat{\mu }$ for various values of ψ held at fixed values to confirm that they are stable with respect to small variations in sampling rates (see Table S4). This indicates that the estimates $\hat{\lambda }$ and $\hat{\mu }$ are reasonable for use in SAASI.

### Transition rates for SAASI

SAASI encodes transitions between discrete regions with a continuous-time Markov chain described by its stochastic rate matrix, Q, similar to existing ancestral character estimation [42] and state-dependent speciation and extinction models [38]. We infer Q for each tree with the ace function in the R package ape [42] and the fitMk function in the R package phytools [43]. Specifically, we consider two models: an equal-rates matrix that models equal transition rates among all states and a hierarchical structure that allows for movements between Blantyre zones to occur at a faster rate than movements between large geographic regions. To obtain estimates in this latter case, we reduce the dimensionality of the matrix Q by grouping the Blantyre zones as a single state and assuming that the transition rates between Blantyre zones are fast relative to the transition rates between Blantyre and the other large regions (Proximal, Global and Global-LSA). We allow for different transition rates between large geographical regions to account for variations in accessibility to other areas. We specify Q so that there are five distinct transition rates: (1) Blantyre to Proximal ($\mu_{1}$), (2) Blantyre to Global and Global-LSA ($\mu_{2}$), (3) Proximal to Global and Global-LSA ($\mu_{3}$), (4) Global to Global-LSA ($\mu_{4}$) and (5) between Blantyre zones ($\mu_{Z}$).

We assume equal transition rates between Zones 1–7 within Blantyre ($\mu_{Z}$) that are a multiple, m, of the transition rates between Global and zones ($\mu_{Z}=m\mu_{2}$). We consider the consequences of different values of m in the Supplement, as well as the impact of assuming equal rates between all regions (see Material S1). The stochastic rate matrix with hierarchical structure that we use in the main SAASI has the form


$$Q=\begin{array}{cc} \begin{array}{cccc} zones & Proximal & Global & Global-LSA \end{array}\\ \begin{array}{c} zones\\ Proximal\\ Global\\ Global-LSA \end{array} & \left(\begin{array}{cccc} M_{Z} & \mu_{1} & \mu_{2} & \mu_{2}\\ \mu_{1} & -(\mu_{1}+{2\mu }_{3}) & \mu_{3} & \mu_{3}\\ \mu_{2} & \mu_{3} & -(\mu_{2}+\mu_{3}+\mu_{4}) & \mu_{4}\\ \mu_{2} & \mu_{3} & \mu_{4} & -(\mu_{2}+\mu_{3}+\mu_{4}) \end{array}\right) \end{array}$$

where $M_{Z}$ is a 7×7 stochastic rate matrix with equal rates ($\mu_{Z}$) on all of the off-diagonal entries.

### Intervention model

**Fig. 2.**
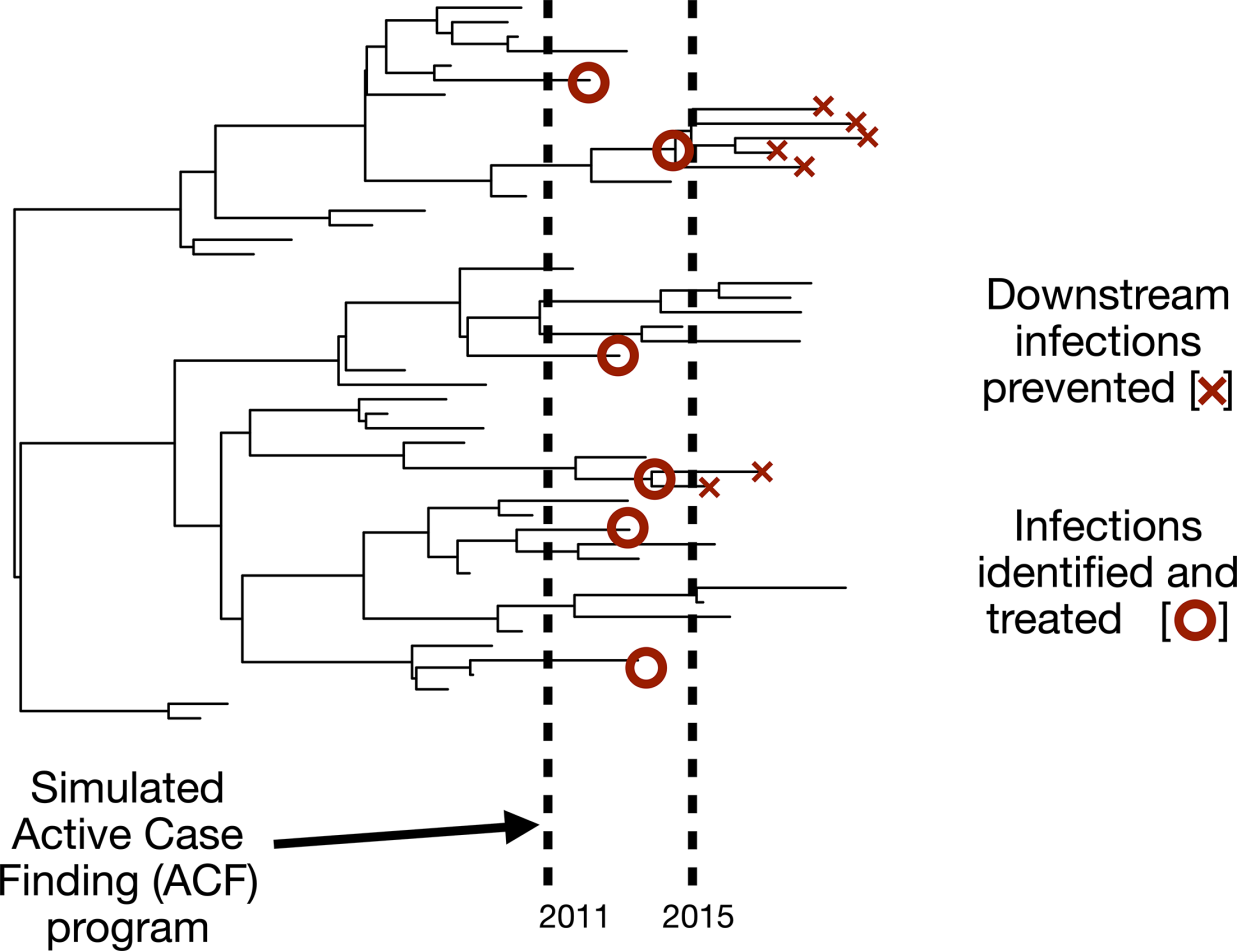
Schematic of ACF intervention to identify additional cases and limit onward transmission. Simulated interventions are specific to particular zones and remove nodes with probability p during a specified time frame. ACF implemented in certain regions may remove nodes with descendants sampled during the time period of data collection (2015–2019).

We simulate ACF interventions by pruning phylogenetic trees in a state-dependent manner that models interventions targeted to particular subsets of zones 1–7 in Blantyre (Fig. 2). To simulate interventions in particular zones, we select a zone, specify a time window and set a probability, p, of identifying and treating cases (represented by active lineages in the trees) before onward transmission occurs. We consider a range of values of the probability of successfully identifying and treating cases before onward transmission (p=0.2,0.4,0.6,0.8, and 1.0). Because ACF usually identifies individuals with TB disease (rather than latently infected individuals), we only consider lineages with a node/tip occurring within 6 months of the end of the ACF intervention window. We call these ‘active lineages’ and allow the possibility that they are identified by ACF.

Once a lineage has been selected for removal, there are two cases to consider: (i) if an active lineage does not have any descendants and instead corresponds to one of the sampled sequences, we remove the edge (but there is no observed indirect benefit of prevented transmissions, because only the tip itself is removed); and (ii) if an active lineage has descendants, we prune the child node corresponding to that edge (and all of its descendants, which models an indirect benefit of prevented transmission). In practice, ACF will directly identify cases that are not ancestral to the sample and may indirectly change detection rates in nearby locations [44,45], so the simulation underestimates direct as well as indirect benefits.

## Results

### Phylogenetic trees for the supplemented datasets

**Fig. 3.**
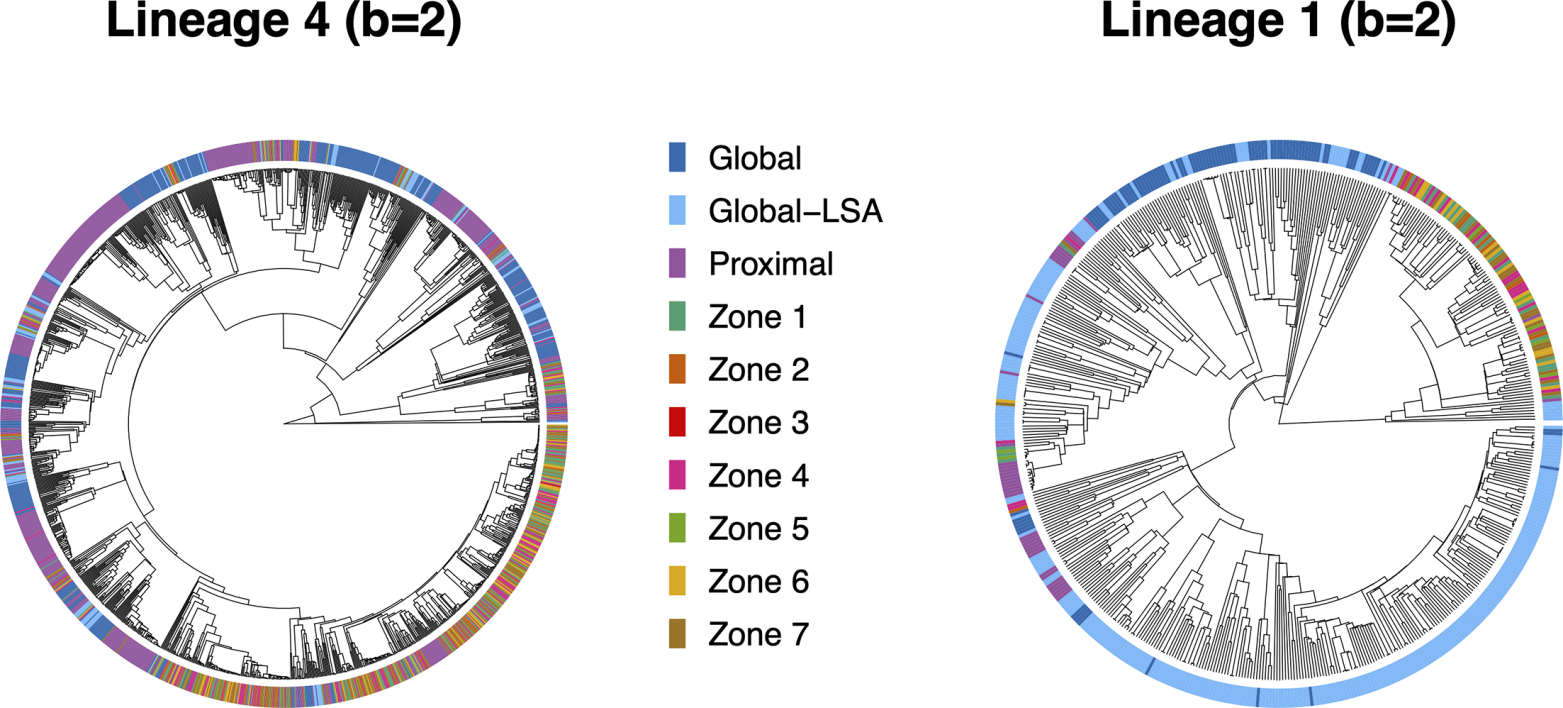
Phylogenetic trees for Lineages 4 and 1 sequences with geographic information. Regions correspond to those shown in Fig. 1, with Global-LSA referring to the regions with low sequence availability (specified in Table S2). Inclusion probability of sequences from Proximal countries is increased by a factor of two relative to their contribution to total notifications because of their proximity to Malawi (*b*=2, see Table 2). See the ‘Methods’ section in the text for descriptions of models used to produce phylogenetic trees.

Most sequences collected from Blantyre, Malawi (Zones 1–7), reside in clades containing small numbers of sequences from Proximal countries and even fewer numbers of Global and Global-LSA sequences, consistent with a substantial amount of transmission localized within national borders (Fig. 3). Blantyre and Proximal sequences surrounded by Global and Global-LSA sequences (located near the eleven o’clock and one o’clock positions in the Lineage 4 tree, for example) might be evidence of recent importation of Mtb into Malawi, but this could merely reflect different amounts of representation of the different sublineages in the data. Phylogenetic reconstructions and locations of regions on the trees relative to each other are robust to variation in the number of sequences included from Proximal countries (Fig. S1), as well as the mean substitution rate in the relaxed molecular clock models, which scales cophenetic distances proportionally (μ=0.5or0.3 SNPs/genome/year; see Fig. S2). Estimates and 95% credible intervals for the standard deviation of the per-branch substitution rates of the relaxed molecular clock models are $\hat{\sigma }=$0.065 (0.057, 0.074) for Lineage 4 with μ=0.3, $\hat{\sigma }=$0.113 (0.099, 0.129) for Lineage 4 with μ=0.5, $\hat{\sigma }=$0.088 (0.081, 0.096) for Lineage 1 with μ=0.3 and $\hat{\sigma }=$0.146 (0.135, 0.159) for Lineage 1 with μ=0.5.

Reconstructed phylogenies for Lineage 1 do not contain sufficient information to identify parameters of the stochastic rate matrix Q using the ace function in R, though the Lineage 4 phylogenies allow for this estimation, under some simplifying assumptions about Q. The data do not contain sufficient information to estimate parameters of more complicated models that account for asymmetric lineage movement between regions. We fit models with a 4×4 symmetric stochastic rate matrix corresponding to transitions among the Blantyre, Proximal, Global and Global-LSA regions (aggregating all of the Blantyre zones together as a single region). The multiplier m describing movement between Blantyre zones relative to movement between the Blantyre and Global regions is not estimable, so we carry out SAASI under different assumed values and examine how results change (m=1,5,10, see Table S3). We use the Lineage 4, b=2 estimates in the SAASI analysis for both lineages (see Material S1 for the estimated rate matrix $\hat{Q}$).

### SAASI

**Fig. 4.**
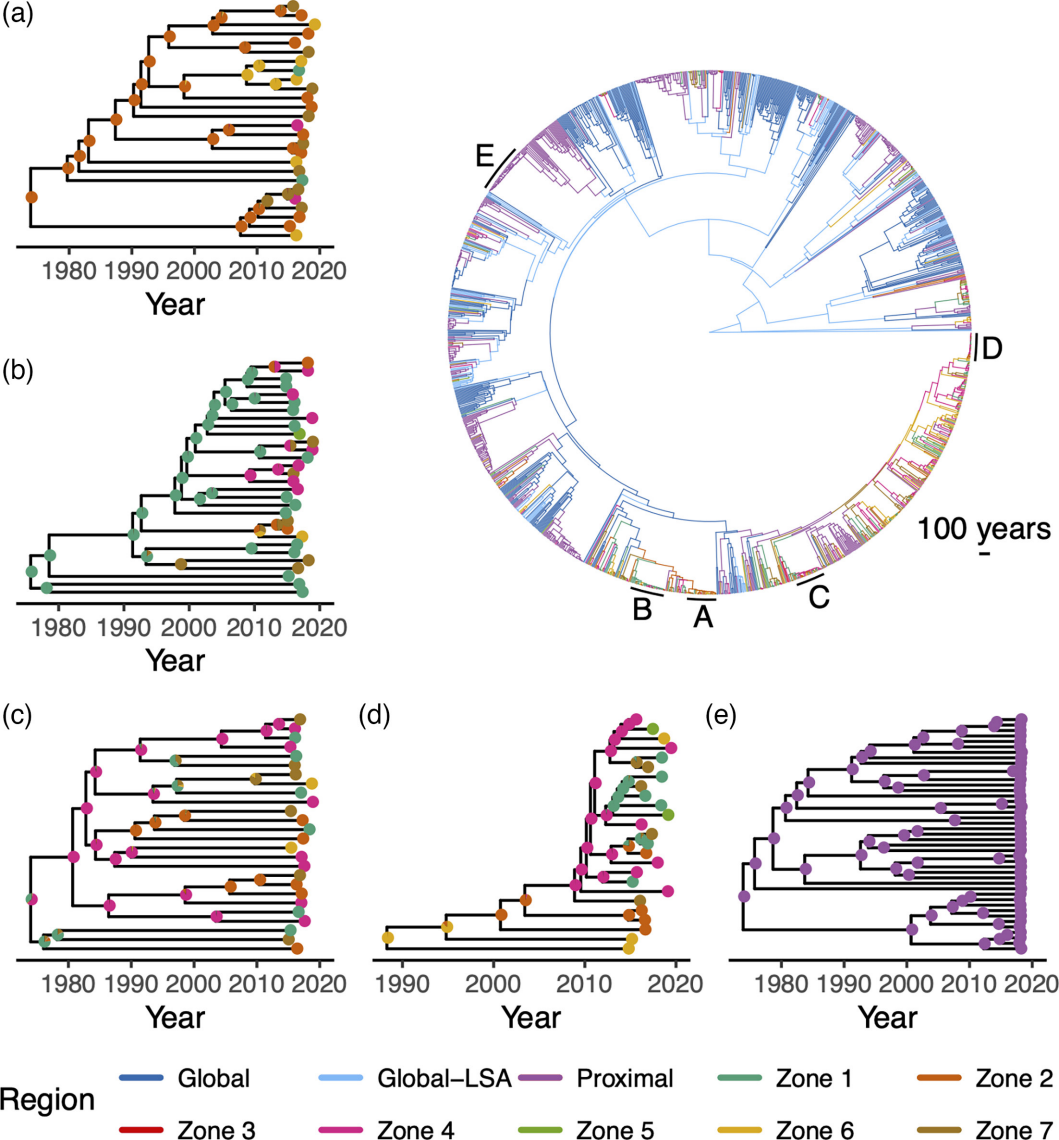
SAASI infers the geographic location of ancestral infections of Lineage 4 Mtb detections across the globe. The tree in the top right shows the ML region of each node inferred by SAASI (edges are coloured by the most likely child node state). Inset trees correspond to the five largest clades with most recent common ancestors (MRCA) occurring since 1970, labelled (a–e) on the Lineage 4 tree. Pie charts in the inset trees show the likelihood of different regions for each ancestral infection, with most indicating a high level of confidence in the ML states. SAASI was carried out for the phylogenetic tree for Lineage 4 in Fig. 3 using parameters in Tables1  2 with *b=2.*

SAASI corroborates the phylogenetic reconstructions (Fig. 3) in that clades of both lineages that have undergone recent, rapid expansion appear to have been circulating primarily within national boundaries since 1990, with evidence of limited dispersal across large geographic regions (Figs4  5). These estimates are stable with respect to changes in the numbers of Proximal sequences included for analysis (Fig. S3).

**Fig. 5.**
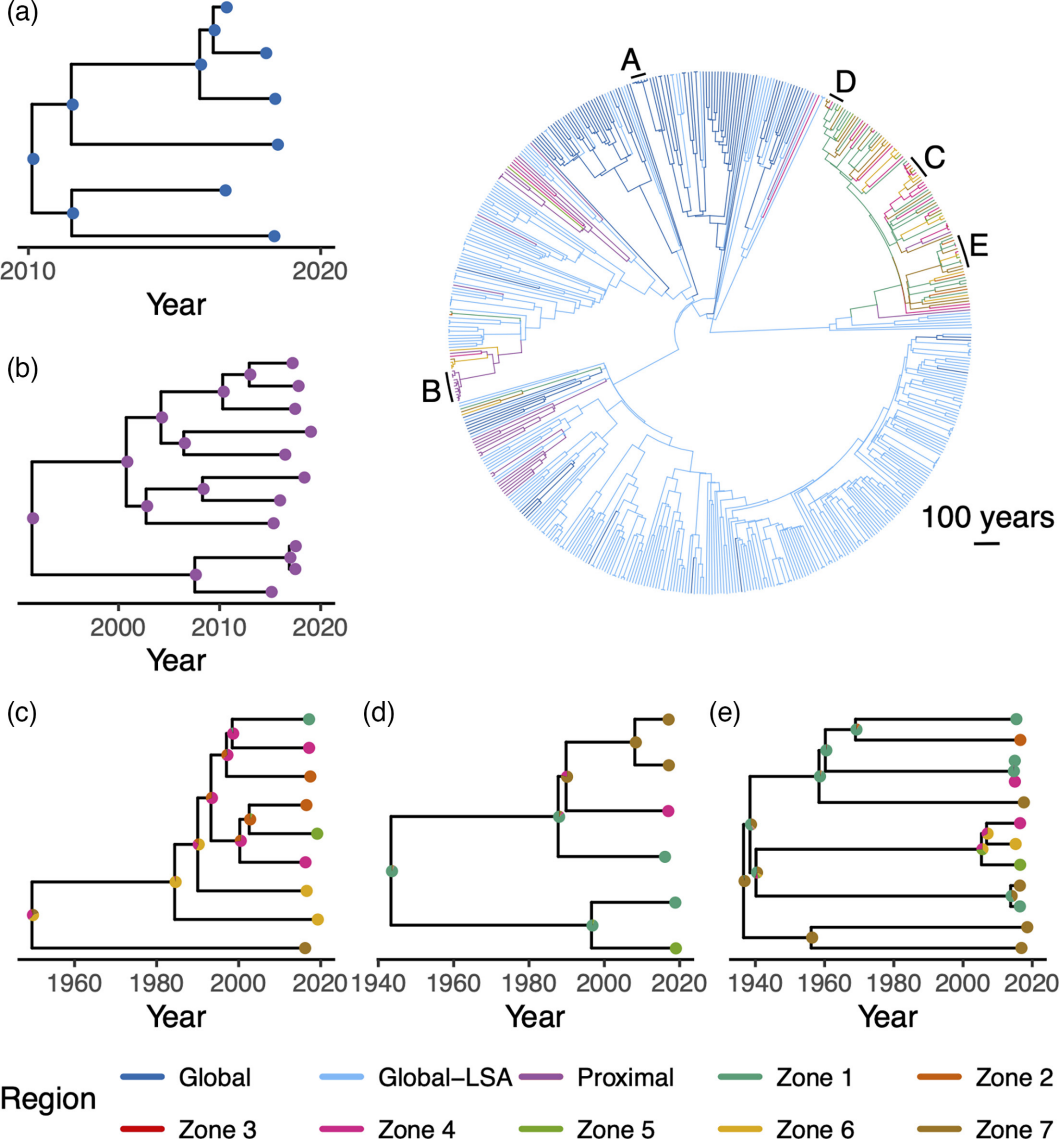
SAASI inferred geographic location of ancestral infections of Lineage 1 Mtb detections across the globe. The tree in the top right shows the ML region of each node inferred by SAASI (edges are coloured by the most likely child node state). Inset trees correspond to the five largest clades with most recent common ancestors (MRCA) occurring since 1920, labelled (a–e) on the Lineage 1 tree. Pie charts in the inset trees show the likelihood of different regions for each ancestral infection, with most indicating a high level of confidence in the ML states. SAASI was carried out for the phylogenetic tree for Lineage one1 in Fig. 3 using parameters in Tables1  2 with *b=2.*

#### Lineage 4

For Lineage 4, SAASI estimates ancestral locations with high confidence in the recent past (back to the late 1970s), but inferences deeper in the trees become sensitive to variation in the stochastic rate matrix governing transitions between states, the diversification rate and the inclusion probability multiplier (b) for Proximal countries (see Figs S4 and S5). Assuming an equal rates model for the stochastic rate matrix governing lineage movements between regions produces very different estimates for the root location of Lineage 4 compared to all the analyses that allow for more rapid movement within Blantyre than between large geographic regions (see parameter sets 1 and 8 in Figs S4 and S5 that infer ‘Proximal’ for root location, in contrast to the rest). Analyses with the model described in the ‘Transition rates for SAASI’ section that accommodates more rapid movement within the city of Blantyre relative to movements between large geographic regions infer either ‘Global-LSA’ or ‘Global’ for the root location under all of the different values of the rate of movement within Blantyre relative to larger regions (m) we considered, as well as the assumption that the diversification rate (λ) is independent of state (Figs 4 and S5; see also Table S3). We also examine the consequences of varying the magnitude of sampling rates ($\psi_{i}$) while holding their relative magnitudes constant and find that estimates for Lineage 4 do not change (Fig. S6).

**Fig. 6.**
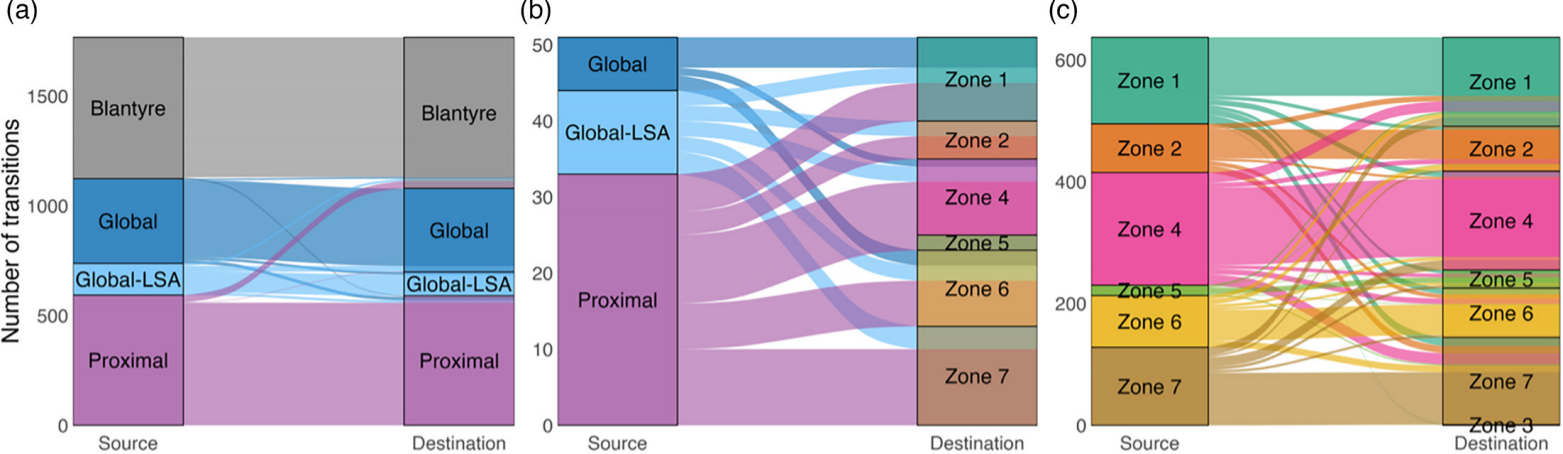
Movement of Lineage 4 between geographic regions from 1999 to 2020 as inferred from SAASI. The alluvial plots display patterns of movement by summarizing observed state changes among parent-child pairs in the phylogenetic trees. The plots are stratified by movement type, including transitions between large geographic regions (**a**), importations into Blantyre from other geographic regions (**b**) and transitions between different zones within Blantyre (**c**). Alluvial plots are generated from the same data in Fig. 4.

Alluvial plots summarizing state transitions in the Lineage 4 phylogenies show that dispersal between large geographic regions is rare, but that movement between zones within Blantyre is much more common (Fig. 6). For Lineage 4, at large geographic scales, parent–descendant pairs in phylogenetic trees belong mostly to the same geographic region, but there are clear instances of importation into Blantyre from other Proximal countries in Africa and other global areas with low sequence availability (Global-LSA). The majority of Lineage 4 importation is attributed to the Proximal region (Fig. 6a, b). Importation into Blantyre appears to be concentrated in Zones 1, 4, 6 and 7, but most zones appear to have experienced at least one importation event (except for Zone 3, which has low numbers of sequences to begin with, Fig. 6b). Within Blantyre, Mtb appears to move between the different zones at a faster rate than larger geographic regions, but many parent/child node pairs reside within the same zone. Movement of Lineage 4 between zones within Blantyre appears mostly uniform, with no obvious flux from a particular subset of zones (Fig. 6c). Inferred locations in the distant past (prior to 1980) are sensitive to variation in the assumed structure of Q, the rate of movement within Blantyre relative to larger regions (m) and the assumption that the diversification rate (λ) is independent of state (Figs S7–S10). Inferences in the more recent past (1980–2020) are more stable by comparison, and variation in m seems to be more important than variation in λ. Estimated numbers of movements do not substantially change if the number of Proximal sequences included is reduced by half (Fig. S11).

#### Lineage 1

**Fig. 7.**
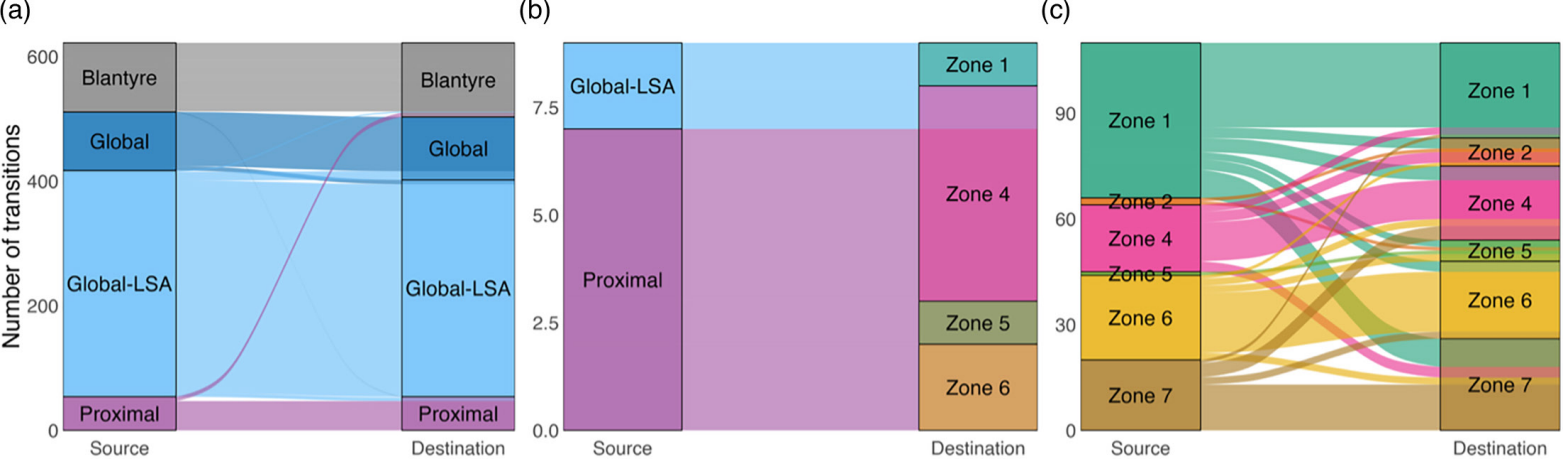
Lineage 1 movement between geographic regions from 1999 to 2020 as inferred from SAASI. The alluvial plots display movements according to the same stratification in Fig. 6. Alluvial plots are generated from the same data in Fig. 5.

For Lineage 1, SAASI identifies small numbers of Mtb movements between geographic regions over the last 100 years and few importations into Blantyre (Fig. 5). SAASI of Lineage 1 appears more robust to variation in the model of lineage movement, the numbers of sequences included from Proximal countries and the overall diversification rate, compared to analyses of Lineage 4 (Figs S12 and S13). Similar to Lineage 4, inferences in the distant past (prior to 1980) are sensitive to variations in SAASI parameters, and different assumed values of m and $\lambda_{G}$ affect estimates in the more recent past (1980–2000; see Figs S12 and S13). The majority of importation into Blantyre during 1999–2020 is attributed to the Proximal, rather than the Global/Global-LSA regions (although the number of inferred importations is small, n=9, Fig. 7). Lineage 1 also shows a different signal of between-zone movement than Lineage 4, with a net flux of Mtb out of Zone 1 into the rest (Fig. 7). Like Lineage 4, inferences in the recent past (1980–2020) are more stable to variation in Q and λ than estimated locations in the distant past (prior to 1980; see Supplemental Figs S14–S17). Estimates of movement from 1990 to 2020 are also robust to variation in the numbers of sequences from Proximal countries included in the analysis (see Fig. S18 with b=1).

#### Asymmetric migration

We investigate the sensitivity of our estimates of Lineage 4 movement with respect to the underlying transition rate matrix, Q. Although the data do not contain sufficient information to estimate asymmetric models, we explore how results from SAASI change in response to different levels of asymmetry in transition rates between the Blantyre zones and the proximal region (the $Q_{1,8}$ and $Q_{8,1}$ terms, both of which equal $\mu_{1}$ in our main analysis). Estimates are stable with respect to increases in the rate of transition from the proximal region into Blantyre ($Q_{1,8}$, Fig. S19), whereas the number of importations from the proximal region into Blantyre decreases as the assumed rate of movement from Blantyre into the proximal region increases ($Q_{8,1}$, Fig. S20). Estimated movements between zones within Blantyre are stable with respect to large changes in these rates (Figs S19–S21).

### Simulated ACF intervention

**Fig. 8.**
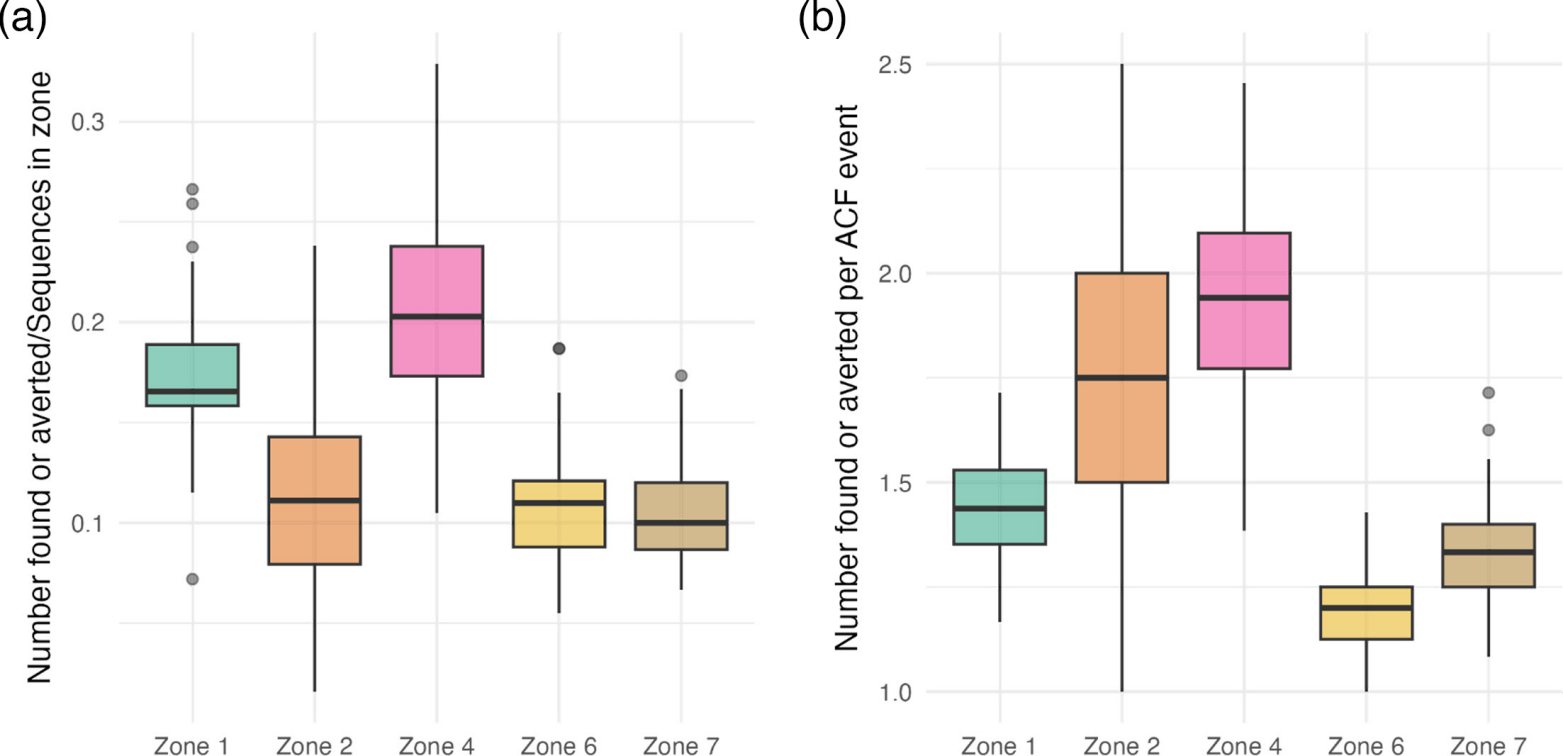
A simulated zone-specific ACF intervention from 2011 through the end of 2014. The impact of ACF is quantified according to the number of cases averted in a particular zone relative to the number of sequences from that zone in the data (**a**) and in terms of the number of cases identified or prevented relative to the number of cases identified through ACF (**b**). In (**b**), the number found or averted includes individuals directly identified through the ACF intervention. Zones 3 and 5 are omitted due to the small number of sequences from those zones in the data. Interventions are simulated using the same data in Figs4  5. The probability of identifying and treating active lineages (p) is set to 40% in this simulation. See the ‘Methods’ section for a description of the intervention simulation.

Fig. 8 shows the impact of simulated zone-specific active case finding interventions carried out between 2011 and the end of 2014. Simulated ACF in Zones 1 and 4 finds or averts the highest number of cases compared to the number of sequences in those zones. This is not strictly the portion of nodes in Zone i found or averted by ACF in Zone i, because some subsequent cases descending from a node in Zone i might occur in another Blantyre zone or another region. This allows us to compare ACF effectiveness across zones with highly variable numbers of infections (Table S1). The number of proximal sequences included in the analysis has a large impact on the model’s results on the effectiveness of ACF (Fig. S22).

In our ACF simulations (which include probabilities of successful identification and treatment before onward transmission ranging from p=0.2 to p=1.0), less than one additional case is prevented, on average (Figs S23 and S24). This is despite a relatively long period over which the impact of ACF is implemented in our simulations: 2011–2014 for the ACF itself, with the removal of onward transmissions until the end of the dataset in 2019. Preventing fewer than one onward transmission per case found is consistent with declining Mtb incidence in Blantyre and also speaks to the somewhat limited potential for ACF to greatly reduce the burden of Mtb, even under optimistic settings (p=1.0; see Figs S23 and S24). However, our approach identifies Zones 2 and 4 as likely to have the highest indirect effect of ACF in terms of onward transmissions prevented and Zones 1 and 4 as likely to have the highest overall effect in terms of cases identified or prevented. However, our results for Zone 4 are not robust to the level of representation of the Proximal region in the data (see Fig. S22). This may be because the distribution of importations from Proximal countries into the zones of Blantyre depends on the numbers of Proximal sequences included in the dataset: in the main analysis (b=2), Zone 4 has many more importations from Proximal countries than Zone 2 (Fig. 6), but the two zones appear to have roughly equal numbers of importations from the Proximal region in the analysis with b=1 (Fig. S11). This results in ACF identifying more individuals in Zone 4 in the b=2 analysis (compared to b=1). These results are robust to the removal of sequences with ambiguous or missing location information (Figs 25 and S26).

## Discussion

Phylogeographic analysis that accounts for sampling differences among locations paves the way for using genomic surveillance data from multiple geographical areas to characterize local and global transmission patterns. We supplemented a genomic epidemiological dataset of Mtb in Blantyre, Malawi, with sequences collected across the world to inform a phylogeographic analysis and investigate the effectiveness of geographically targeted interventions to reduce transmission within Blantyre. Our analysis suggests that importation of Mtb into Blantyre is rare relative to transmission occurring within Blantyre (51/1767 inferred state transitions on the Lineage 4 phylogeny during the time period 1990 to 2020 are movements into Blantyre, and 9/622 of the inferred state transitions on the Lineage 1 phylogeny during the same time period are movements into Blantyre) and that the relatively small number of movements into Blantyre originate predominantly from nearby countries. This includes countries that contribute to substantial land-based travel across the Malawi border. Our phylogenetic reconstructions contain large clades that consist entirely of sequences collected within Blantyre, as well as large clades consisting entirely of sequences from other regions, which is consistent with previous analyses that have identified clear spatial localization of particular lineages (or sublineages) of Mtb in Africa and elsewhere [46].

We paired our analysis with a simple simulation study that considers ACF interventions carried out in specific zones within Blantyre from 2011 through the end of 2014. Cluster-randomized trials have not detected transmission reductions following ACF, and our results indicate that estimating transmission reductions in Blantyre following interventions is likely to be complicated by unobserved movement patterns within and across the borders of Malawi. Our results suggest that ACF might be moderately more effective at reducing transmission when targeted toward particular zones of Blantyre (Zones 2 or 4), but these conclusions are sensitive to the number of sequences from Proximal countries included in our dataset (compare Figs 8 and S22). Our simulations also show that the impact of ACF is rather modest, with less than one onward infection prevented per case found, on average (Fig. 8). This is consistent with the fact that TB incidence is declining in Malawi. In addition, the results of our simple simulations are highly variable, particularly for Zones 2 and 4. Zone 4 corresponds to Ndirande, which is a very densely populated area of the city: there is a busy market, and people often move to Ndirande from rural areas to set up small businesses as they establish themselves in the city. Zone 2 corresponds to Likhabula and is a mixture of newer peri-urban informal settlements and more established peri-urban and urban settlements. Overall, there are fewer municipal services, and access to healthcare is more limited compared to the rest of the city. It is possible that a higher sequencing fraction (18% of notified cases in Blantyre were sequenced in this analysis) or a longer time frame would lead to lower variation in the simulated impacts of ACF or that the overall simulated impacts would be different.

A strength of our approach is that we can account for both direct benefits (number of infections identified) and indirect benefits (number of onward infections prevented) over a defined period of time. A limitation is that we can only quantify these effects relative to sequenced infections captured in the data. This accounts in part for the wide ranges in the impacts of our simulated interventions and motivates our choice of impact measures (relative to the number of sequences in each zone and relative to the number of cases found). In our ACF simulations, we optimistically assume that cases will not become infected by different transmission routes if their infection is prevented, and we do not compare different types of interventions. We also do not account for changes in case detections resulting from interventions like ACF, which can occur in the particular location where an intervention is active, as well as neighbouring areas [44,45].

In a declining epidemic like that of TB in Blantyre [4], it seems reasonable that infections prevented through ACF will not be infected by an alternate transmission pathway, but localization of transmission in space or within risk groups could undermine the validity of this assumption. In particular, the use of antiretroviral therapy (ART) and isoniazid for HIV positive individuals slows progression from latent to active TB, reducing notification rates and probably transmission as well [47]. We do not account for these effects in this analysis, but a substantial fraction of TB notifications in Blantyre are HIV positive individuals taking ART and isoniazid [22], and interventions to reduce transmission could have different impacts on this risk group compared to the rest of the population [48,49]. In our analysis, we assume that nodes in the phylogeny correspond to infectious individuals that can be identified through ACF screening. If this assumption does not hold in reality [47,48], then our simulations may overestimate reductions in TB (cf. Fig. 8). A large force of infection attributable to individuals not captured by these data could also mean that infections downstream of a prevented infection have a high probability of becoming infected by someone else.

In augmenting the Blantyre data with publicly available sequences from ENA, we ensure that the additional sequences belong to the same sublineages present in Blantyre. Our analysis of the data with enriched representation from the proximal region (our *b*=2 analysis) includes all of the publicly available sublineage 4.3 sequences available from the proximal region. Sublineage 4.3 is the most prevalent in Blantyre, and our finding that the majority of importations into Blantyre come from the proximal region is consistent with similar sublineage compositions of those regions. Different sublineages present in these data could introduce variation into the substitution rate, but the sampling period of our data (2015—2019) is too short to reliably estimate the substitution rate in a molecular clock model [37]. We use fixed mean substitution rates consistent with previously reported estimates obtained from very large datasets for Lineage 4 [36], but estimate the standard deviation of the per-branch substitution rates (σ) with BactDating. Although our estimates for the variation in the per-branch substitution rates are low (see the ‘Results’ section), the limited timeframe of these data may obscure variation in the substitution rate by sublineage and/or over time. Uncertainty in the substitution rate itself does not appear to introduce uncertainty in our overall results because it has the effect of scaling overall branch lengths while preserving relative branch lengths within the phylogenies (Fig. S2). Estimated rates in Markov models fit to the phylogenies (using ace or fitMk) offset changes in absolute scaling introduced by changes in the substitution rate, with the consequence that our phylogeographic inferences are robust to changes in the overall substitution rate (at least over the range μ=0.3to0.5 SNPs/genome/year). There may have been changes in the substitution rate over time, but we focus on geographic movements in recent decades that are unlikely to be impacted by variations in clock rate (and hence edge lengths) deep in the phylogenies.

Our inferences of Mtb movement and impacts of geographically targeted interventions are rooted in phylogeographic reconstructions that use SAASI to infer the geographic locations of internal nodes of phylogenetic trees. SAASI has the advantage that it can account for sampling differences (overcoming a major challenge for phylogeography), but it requires several inputs that must be independently estimated. Among these is a stochastic rate matrix governing the Markovian model of lineage movement among locations (Q, which we estimate using ‘ace’ in the ‘ape’ package [42] in R). Assumptions about the structure of Q can greatly affect the results of phylogeographic analyses in general [50], and ours is no exception. Some analyses jointly estimate Q alongside phylogenetic trees in a Bayesian framework (sometimes incorporating stochastic variable selection, particularly when Q is sparse [51]). However, these methods do not account for sampling differences by location, which is an important source of bias when present [23], and they do not scale well to large datasets [52]. SAASI uses a fixed phylogenetic tree and is tractable for analysing a large number of sequences, falling under the category of discrete trait analyses of infectious disease phylogeography (contrasted with phylodynamics) [52]. SAASI also depends on other fixed inputs, including state-dependent sampling rates ($\psi_{i}$) and an overall diversification rate (λ). The underlying birth-death-sampling model is simplistic, even with adaptations for sampling variation over time and by location. Despite these limitations, SAASI improves upon standard methods for analysing large-scale datasets [24], and the estimates we report for the migration history of Mtb in Blantyre in the recent past (1980–2020) are robust to variation in Q, $\psi_{i}$, and λ (see Material S1). A limitation of our analysis is the paucity of data from locations in Malawi other than Blantyre during the time period 2015—2019, which prevents us from reconstructing lineage movements between different cities or regions within Malawi. Future work could include data that have been collected from the Karonga District in Malawi from 1995 to 2014, but would need to develop more complex models of lineage movement while accounting for a different time period of data collection than the data analysed in this study (2015 to 2019) [21,53]. There are substantial amounts of migration between rural and urban areas within Malawi related to work; seasonal farming and fishing activities contribute to movements within the borders of the country [54,55], and it is likely that this affects the movement of Mtb lineages within Malawi.

Coupling large genomic surveillance datasets with new methods for phylogenetic and phylogeographic analyses can reveal movements of pathogens by leveraging information at large and small spatiotemporal scales. Modelling approaches ranging from the simple intervention model we used to richer dynamic transmission models fit to longitudinal data can inform projections of the likely impact of interventions such as spatially targeted ACF. In addition to collecting data within a particular country or region of interest, data from the rest of the world are useful and important for characterizing pathogen movement within and across borders, and this information is essential for public health practitioners seeking to reduce the prevalence of a disease like Mtb within their jurisdictions.

## Supplementary material

10.1099/mgen.0.001674 Uncited Supplementary Material 1.

## Abbreviations

ACF: active case finding
ART: antiretroviral therapy
BiSSE: binary state-dependent speciation and extinction
ENA: European Nucleotide Archive
ML: maximum likelihood
Mtb: *Mycobacterium tuberculosis*
SAASI: sampling-aware ancestral state inference
TB: tuberculosis
WHO: World Health Organization
