# Supplementary material for: Genomic epidemiology of Mycobacterium tuberculosis in Malawi: using global phylogeography to understand the impact of geographically focused interventions
Source: Microb Genom. 2026 Apr 8;12(4):001674. doi: 10.1099/mgen.0.001674 (PMC13060038; doi:10.1099/mgen.0.001674)
Supplement: Uncited Supplementary Material 1. [file mgen-12-01674-s001.pdf]

Supplementary material for: Genomic epidemiology of Mycobacterium tuberculosis in Malawi: using global phylogeography to understand the impact of geographically-focused interventions

Authors

Alexander B. Beams[1*](ORCID: 0000-0002-6656-0217), Yexuan Song[1](ORCID: 0009-0004-8316-4293), Jennifer McNichol[1](ORCID: 0000-0002-7100-0855), Bradley R. Jones[1] (ORCID: 0000-0003-4498-1069), Benjamin Sobkowiak[2](ORCID: 0000-0002-1382-1137), Peter MacPherson[3,4,5](ORCID: 0000-0002-0329-9613), Marriott Nliwasa[6,7](ORCID: 0000-0002-3100-5512), Victor Ndhlovu[8] (ORCID: 0000-0003-1606-0959), Mphatso D. Phiri[4,8,9] (ORCID: 0000-0002-4072-9715), Ted Cohen[10](ORCID: 0000-0002-8091-7198), Caroline Colijn[1] (ORCID: 0000-0001-6097-6708)

Affiliations

[1] Department of Mathematics, Simon Fraser University

[2] Infection, Immunity and Inflammation Dept, University College London

[3] School of Health & Wellbeing, University of Glasgow

[4] Malawi Liverpool Wellcome Programme, Blantyre, Malawi

[5] Clinical Research Department, London School of Hygiene & Tropical Medicine

[6] Division of Epidemiology and Biostatistics, School of Public Health, Faculty of Health Sciences, University of the Witwatersrand, Johannesburg, South Africa

[7] Helse Nord Clinical Research and Training Initiative, Kamuzu University of Health Sciences, Blantyre, Malawi

[8] Kamuzu University of Health Sciences, Blantyre, Malawi

[9] Liverpool School of Tropical Medicine, Liverpool, United Kingdom

[10] Department of Epidemiology of Microbial Diseases, Yale School of Public Health, Yale University

Corresponding author and email address

*Corresponding author: Alexander B. Beams (abeams@sfu.ca)

Data

There are 9 individuals with Lineage 4 infection for whom zone information is not available. We assign one individual to Zone 2 based on the admitting hospital. Three others are diagnosed at Queen Elizabeth (QE) Central Hospital; one of these also has an admission to Mwaiwathu hospital (in same zone as QE central, Zone 5); we assigned these individuals to Zone 5. We assigned the 5 remaining Lineage 4 infections without zone information to randomly assigned Zones based on the number of samples in each. Similarly, 4 individuals infected with Lineage 1 lacked zone information, and we randomly assigned these Zones weighted by the number of Lineage 1 detections. We also examine how results change by dropping these datapoints from the simulated intervention (Supplemental Figure 25 and Supplemental Figure 26).

Table S1: Number of sequences from each zone of Blantyre for Lineages 4 and 1.

Region	Lineage 4	Lineage 1
Zone 1	114	25
Zone 2	56	7
Zone 3	1	
Zone 4	120	23
Zone 5	27	7
Zone 6	74	17
Zone 7	126	24

Table S2: Composition of the augmented dataset from ENA. Column one lists each country for which we have sequences from either Lineage 1 or Lineage 4. Column two shows which region (Global, Global-LSA, Proximal) each country belongs to in our analysis. Note that Indonesia is included in “Global" for the Lineage 4 analyses and included in “Global-LSA" for Lineage 1 analyses. Columns three and four show the number of sequences we have used for Lineage 4 and Lineage 1 analyses, respectively. See Table 6 for the number of sequences from each zone of Blantyre.

Country	Region category	Lineage 4	Lineage 1
Argentina	Global	4	
Azerbaijan	Global	1	
Bangladesh	Global-LSA	12	4
Belarus	Global	1	

Country	Region category	Lineage 4	Lineage 1
Botswana	Proximal	1	
Brazil	Global	33	
Bulgaria	Global	1	
Cambodia	Global		20
China	Global	64	3
Colombia	Global	6	
Cote d'Ivoire	Global	9	
Dem. Rep. Congo	Proximal	3	
Ecuador	Global	1	
Ethiopia	Global	38	1
France	Global	1	
Georgia	Global	1	
Ghana	Global	6	
India	Global-LSA	75	263
Indonesia	Global	107	
Indonesia	Global-LSA		25
Iran	Global	1	
Italy	Global	1	
Japan	Global	1	
Kazakhstan	Global	1	
Kenya	Proximal	6	
Madagascar	Global	7	6
Malaysia	Global		15
Mexico	Global	1	
Morocco	Global-LSA	2	
Myanmar	Global	4	22
Nepal	Global-LSA	1	
Nigeria	Global	17	
Pakistan	Global-LSA	10	18
Papua New Guinea	Global	2	
Paraguay	Global	1	
Peru	Global	12	
Poland	Global	2	
Rep. Moldova	Global	1	

**Country**	**Region category**	**Lineage 4**	**Lineage 1**
Romania	Global	5	
Rwanda	Proximal	86	
Saudi Arabia	Global-LSA		1
South Africa	Proximal	309	20
South Korea	Global	2	
Sri Lanka	Global	2	
Taiwan	Global	1	
Tanzania	Proximal	23	19
Thailand	Global	2	7
Timor-Leste	Global	1	1
Tunisia	Global	1	
Uganda	Proximal	36	2
Ukraine	Global	2	
UK	Global	1	
USA	Global	2	1
Vietnam	Global	3	17

Table S3: Parameter variations for SAASI. Variations in parameters are considered for b, the multiplier for inclusion probability of sequences from Proximal countries, the form of the stochastic rate matrix, Q, governing lineage transitions between different locations, the multiplier, m, describing transition rates between Blantyre zones relative to the transition rate between Blantyre and Global regions (μ_2), and an increased diversification rate in the Global state (λ_G). For Q structure, "hierarchical" refers to the block matrix form described in Section 2.7 of the main text. Values of $\hat{\lambda}$ are in Table 1.

Parameter set	b	Q structure	m	λ_G	
				Lineage 1	Lineage 4
set01	1	equal rates	1	0.004	0.005
set02	1	hierarchical	1	0.004	0.005
set03	1	hierarchical	5	0.004	0.005
set04	1	hierarchical	10	0.004	0.005
set05	1	hierarchical	1	0.04	0.05
set06	1	hierarchical	5	0.04	0.05
set07	1	hierarchical	10	0.04	0.05
set08	2	equal rates	1	0.006	0.004

Parameter set	b	Q structure	m	λ_G	
set09	2	hierarchical	1	0.006	0.004
set10	2	hierarchical	5	0.006	0.004
set11 (main)	2	hierarchical	10	0.006	0.004
set12	2	hierarchical	1	0.06	0.04
set13	2	hierarchical	5	0.06	0.04
set14	2	hierarchical	10	0.06	0.04

Results

We estimate a symmetric stochastic rate matrix by fitting a Markov chain with four states (Blantyre, Proximal, Global, and Global-LSA) to the Lineage 4, $b = 2$ data using the ace function in R. The Lineage 1 phylogenies do not contain sufficient information to estimate a model, so we use the inferred matrix for Lineage 4 for all analyses. For SAASI, we augment the 4×4 matrix to a 10-dimensional symmetric stochastic rate matrix by setting movements between the seven Blantyre zones to a multiple of the estimated rate between the Blantyre and Global regions ($\mu_Z = m\mu_2$). The resulting matrix is given by

$$\hat{Q} = \begin{array}{c} \\ zones \\ Proximal \\ Global \\ Global - LSA \end{array} \begin{array}{cccc} zones & Proximal & Global & Global - LSA \\ \left(\begin{array}{c} M_Z \\ 6 \times 10^{-4} \\ 1 \times 10^{-4} \\ 1 \times 10^{-4} \end{array}\right. & \begin{array}{c} 6 \times 10^{-4} \\ -1.2 \times 10^{-4} \\ 3 \times 10^{-4} \\ 3 \times 10^{-4} \end{array} & \begin{array}{c} 1 \times 10^{-4} \\ 3 \times 10^{-4} \\ -8 \times 10^{-4} \\ 4 \times 10^{-4} \end{array} & \left.\begin{array}{c} 1 \times 10^{-4} \\ 3 \times 10^{-4} \\ 4 \times 10^{-4} \\ -8 \times 10^{-4} \end{array}\right) \end{array}$$

where the off-diagonal terms of M_Z are all equal to $\mu_Z = m\mu_2$, $\mu_2 = 1 \times 10^{-3}$ (we use $m = 10$ in the main analysis). The rates are in units of year^{-1}.

We consider the effect on SAASI of changing the numbers of Proximal sequences included in the data ($b = 2$ or $b = 1$), variations in the stochastic rate matrix (Q) governing lineage movements between geographic regions, and we explore how results change if the diversification rate for the Global region (λ_G) is larger than the others (λ). In general, ancestral inferences deeper in the phylogenetic trees (longer than 50-100 years ago) are sensitive to these variations (Supplemental Figure 4 – Supplemental Figure 18), so we do not report results for inferred migration events happening in the distant past in the main results. Variations in the magnitude of the sampling rates (ψ_i) do not noticeably affect results (Supplemental Figure 6), but relative differences in rates are important.

Table S4: Parameter estimates $\hat{\lambda}$ and $\hat{\mu}$ for different values of ψ. All rates are reported in units of year^{-1}.

	Lineage 4, $b = 1$		Lineage 4, $b = 2$		Lineage 1, $b = 1$		Lineage 1, $b = 2$	
ψ	$\hat{\lambda}$	$\hat{\mu}$	$\hat{\lambda}$	$\hat{\mu}$	$\hat{\lambda}$	$\hat{\mu}$	$\hat{\lambda}$	$\hat{\mu}$
10^{-3}	1.9×10^{-3}	1.0×10^{-3}	2.7×10^{-3}	1.0×10^{-3}	3.0×10^{-3}	1.0×10^{-3}	3.2×10^{-3}	1.0×10^{-3}
10^{-4}	9.4×10^{-2}	8.6×10^{-2}	9.9×10^{-2}	8.7×10^{-2}	9.3×10^{-2}	7.6×10^{-2}	9.2×10^{-2}	7.5×10^{-2}
10^{-5}	9.4×10^{-1}	9.3×10^{-1}	9.9×10^{-1}	9.9×10^{-1}	9.3×10^{-1}	9.1×10^{-1}	9.2×10^{-1}	9.0×10^{-1}
10^{-6}	9.4×10^{0}	9.3×10^{0}	9.9×10^{0}	9.8×10^{0}	9.3×10^{0}	9.2×10^{0}	9.2×10^{0}	9.1×10^{0}

Results for more recent time periods (since 1990) are relatively stable to these perturbations, and broadly corroborate the view that most transmission is localized within national boundaries. In particular, our estimates for movements between large geographic regions, importations into Blantyre, and movements within Blantyre for the time period 1990–2020 are robust to variation in the number of sequences included from Proximal countries (Supplemental Figure 7 – Supplemental Figure 10 and Supplemental Figure 14 – Supplemental Figure 17). However, zone-specific effectiveness of simulated ACF interventions in different zones of Blantyre varies considerably with the number of Proximal sequences included the analysis (Supplemental Figure 22).

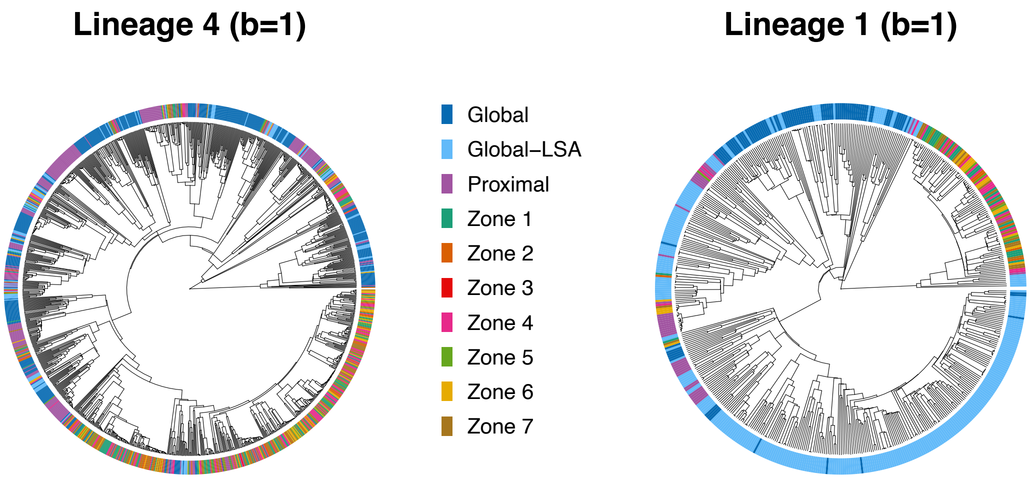

Supplemental Figure 1: Phylogenetic trees for Lineages 4 and 1 sequences with sequences from Proximal regions sampled with probability equal to their contribution to total TB notifications ($b = 1$). Regions correspond to those shown in Figure 1, with Global-LSA referring to the regions with low sequence availability (specified in Table S2). See Methods in the text for descriptions of models used to produce phylogenetic trees.

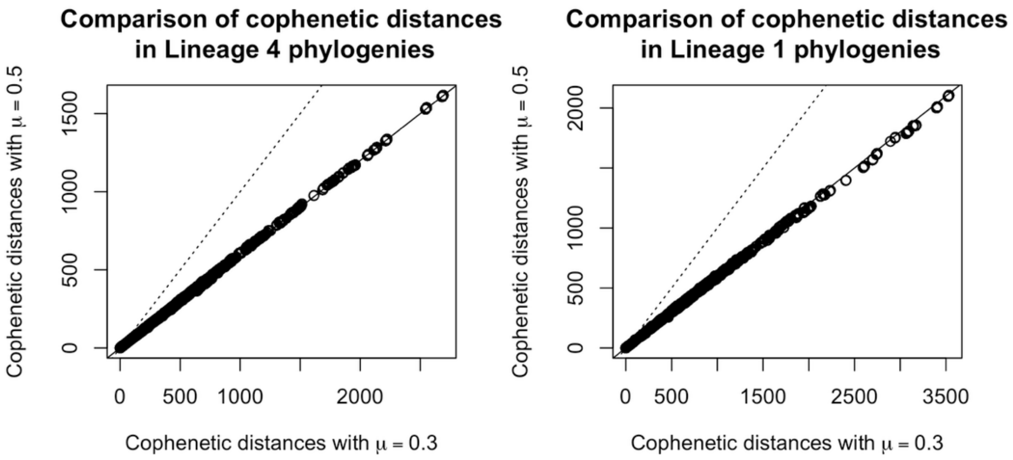

Supplemental Figure 2: Comparison of cophenetic distances in Lineage 4 (left) and Lineage 1 (right) phylogenies under different mean substitution rates in BactDating ($\mu = 0.5$ or 0.3 SNPs/genome/year). The dotted line in each figure is the 1-1 line, and the solid line through the datapoints in each figure passes through the origin with slope 5/3. The phylogenies are obtained from 10^6 MCMC iterations in BactDating with the mean substitution rate fixed, but the standard deviation of the per-branch substitution rates (σ) estimated.

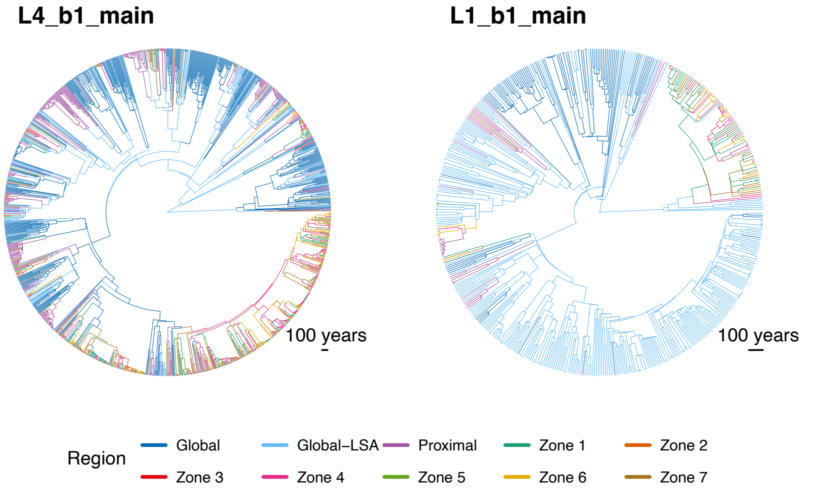

Supplemental Figure 3: SAASI trees for Lineage 4 and Lineage 1 under the same model parameterization but with fewer Proximal sequences than the analysis in the main text ($b = 1$). Regions correspond to those shown in Figure 1, with Global-LSA referring to the regions with low sequence availability (specified in Table S2). Inclusion probability of sequences from Proximal countries equals their expected contribution to total notifications (b=1, see Table *2). See Methods in the text for descriptions of models used to produce phylogenetic trees.*

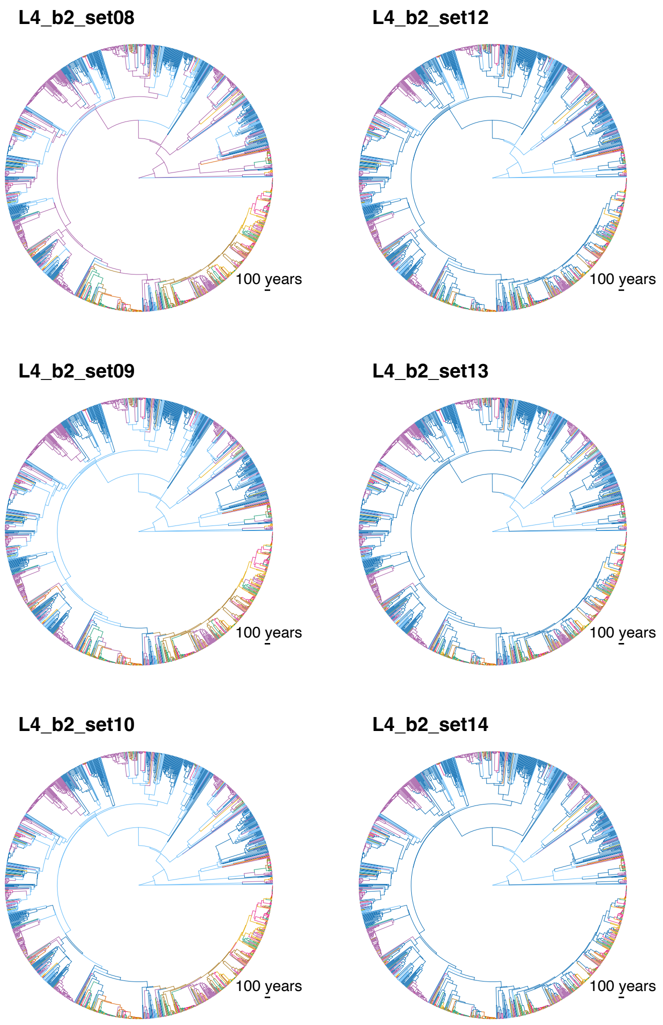

Supplemental Figure 4: SAASI trees for Lineage 4 under alternative parameterizations for the model with the same number of sequences from Proximal countries as in the main text ($b = 2$). Parameter sets correspond to those in Table S3. In addition to an equal rates model for the stochastic rate matrix (Q) describing lineage movements (parameter set 8), parameter variations include an elevated diversification rate in the Global region (λ_G) and different multipliers for movement within Blantyre relative to the Global-to-Blantyre transitions (m). In the left column ($\lambda_G = 0.004$), the rows correspond to an equal-rates model for Q, and hierarchical structures with Blantyre multipliers of $m = 1$ and 5; the right column ($\lambda_G = 0.04$) has hierarchical structure in Q with Blantyre multipliers of $m = 1, 5,$ and 10 down the rows. The analysis in Figure 4 uses $\lambda_G = 0.004$, Blantyre multiplier equal to $m = 10$ with a hierarchical Q matrix (parameter set 11). Colors correspond to the same locations as in Supplemental Figure 1.

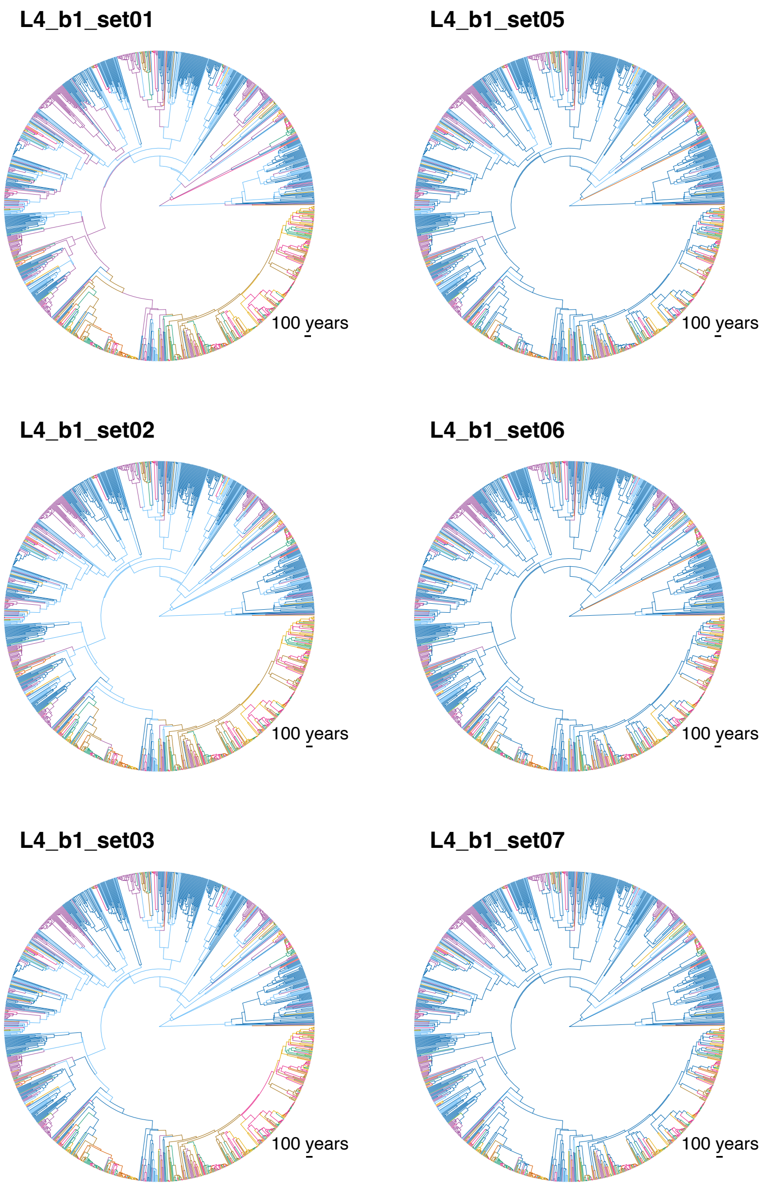

Supplemental Figure 5: SAASI trees Lineage 4 under alternative parameterizations for the model with fewer Proximal sequences than are included in the main text ($b = 1$). Parameter sets correspond to those in Table S3. In addition to an equal rates model for the stochastic rate matrix (Q) describing lineage movements (parameter set 1), parameter variations include an elevated diversification rate in the Global region (λ_G) and different multipliers for movement within Blantyre relative to the Global-to-Blantyre transitions (m). In the left column ($\lambda_G = 0.005$), the rows correspond to an equal-rates model for Q, and hierarchical structures with Blantyre multipliers of $m = 1$ and 5; the right column ($\lambda_G = 0.05$) has hierarchical models with Blantyre multipliers of $m = 1$, 5, and 10. Colors correspond to the same locations as in Supplemental Figure 1.

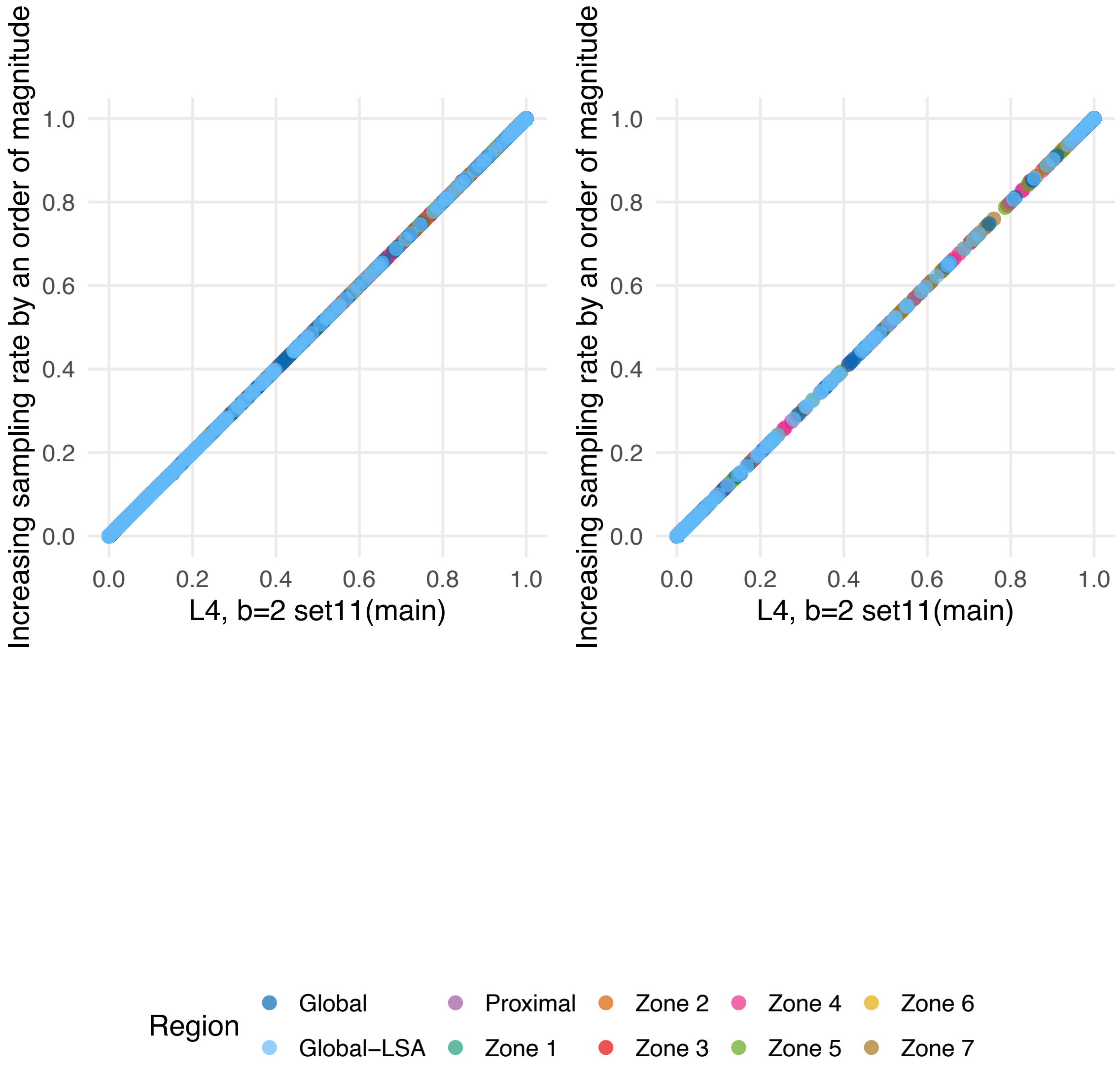

Supplemental Figure 6: Comparison between ancestral state inference results of Lineage 4 main analysis and increasing the sampling rates ψ_i by a factor of 10. (A) Inferred ancestral states probabilities from $t = 0$ to $t = T_{MRCA}$ for the analysis in the main text (main analysis) and increasing the sampling rates. (B) Inferred ancestral states probabilities from 1980 to 2020 in the main analysis and under the adjustment.

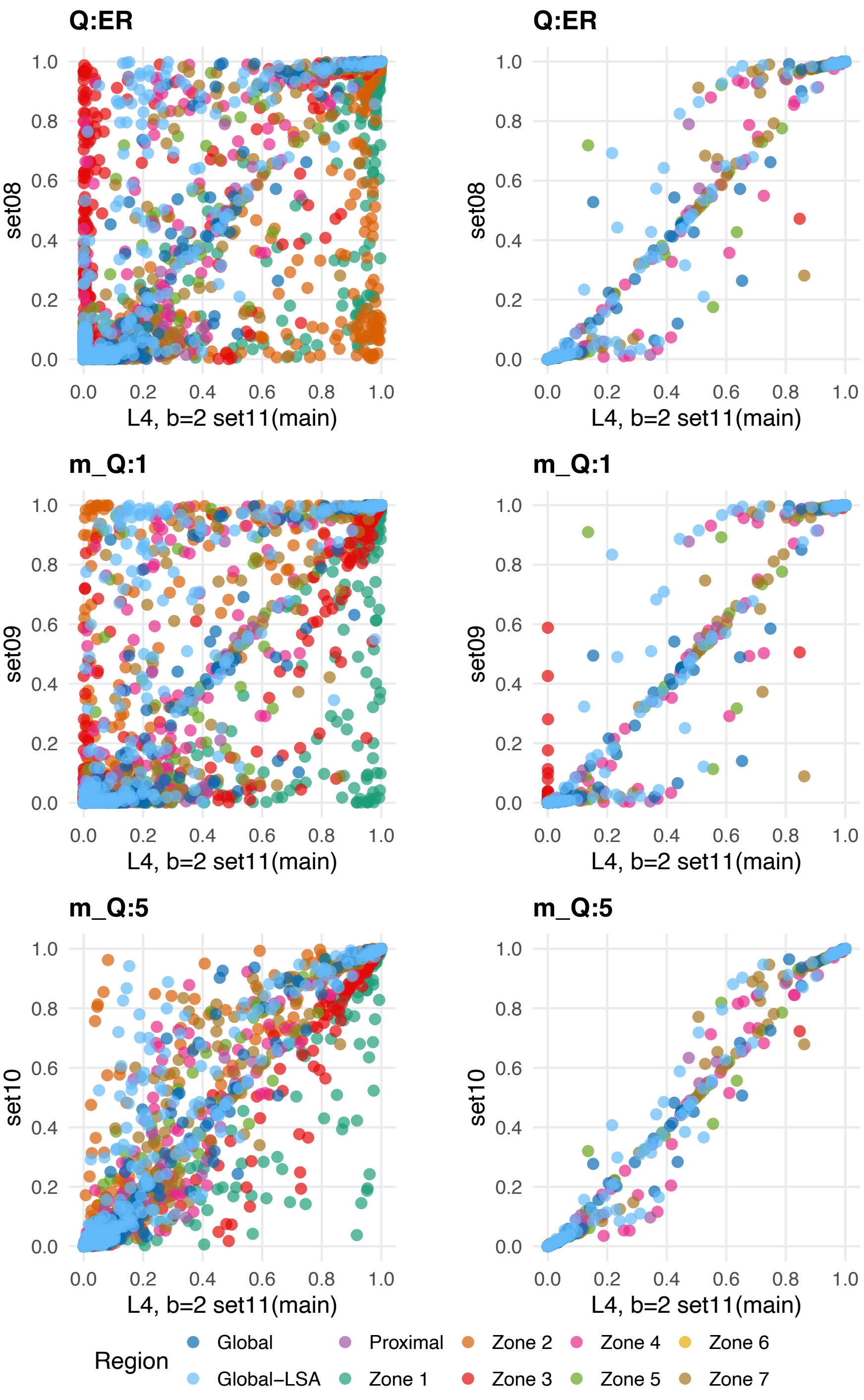

Supplemental Figure 7: Comparison between SAASI results of Lineage 4, $b = 2$, parameter set 11 (main analysis) and modifications of Q under a diversification rate independent of state ($\lambda_G = 0.004$, see Table S3). The left column shows inferred ancestral state probabilities from $t = 0$ to $t = T_{MRCA}$. The right column shows inferred ancestral state probabilities from 1980 to 2020. Q:ER: equal rates matrix; m_Q: multiplier m for transition rates between Blantyre zones (μ_Z) relative to Blantyre–to–Global transition rates (μ_2) in the hierarchical Q model.

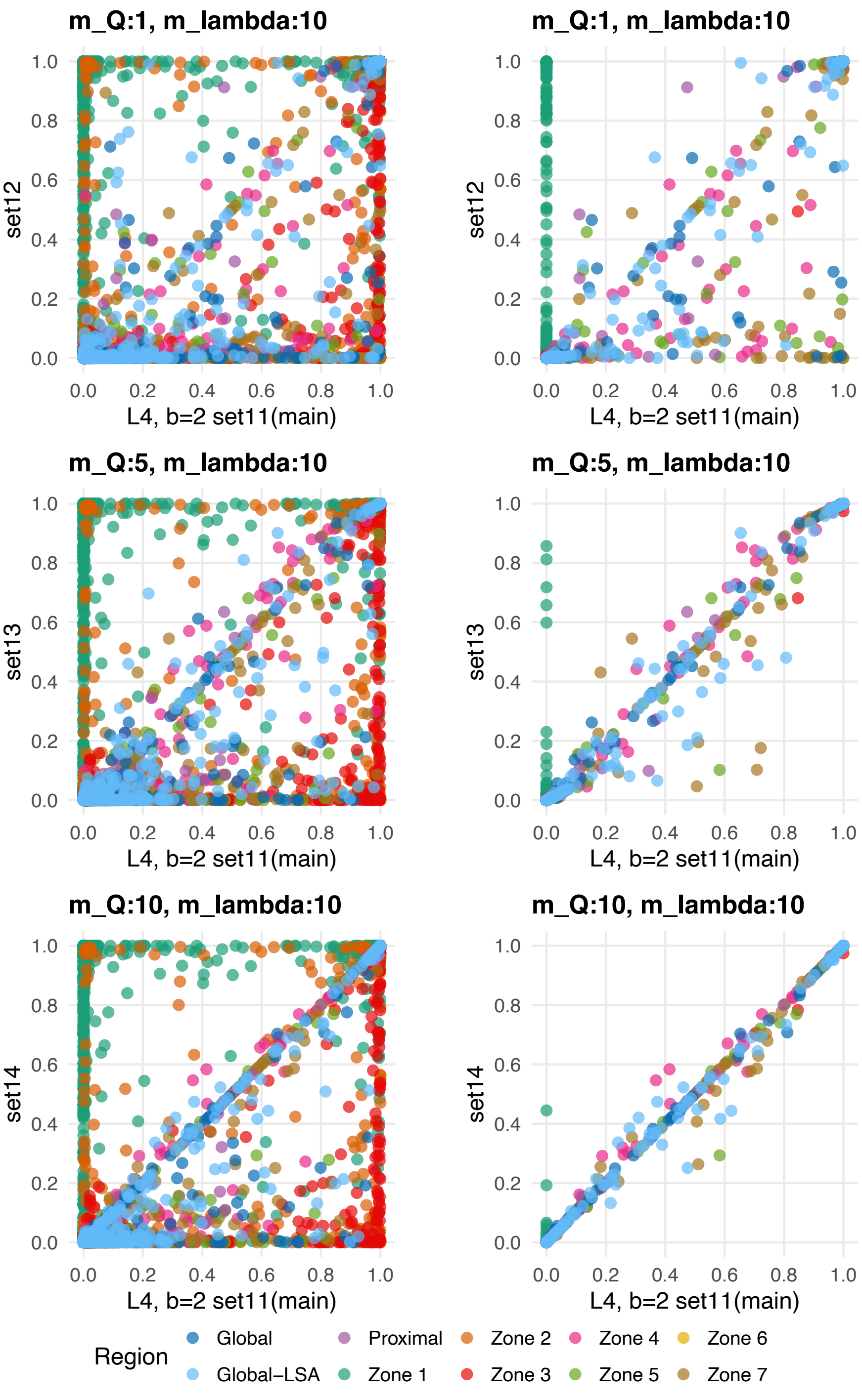

Supplemental Figure 8: Comparison between SAASI results of Lineage 4, $b = 2$, parameter set 11 (main analysis) and modifications of Q under an elevated diversification rate for the Global state ($\lambda_G = 0.004$, see Table S3). The left column shows inferred ancestral state probabilities from $t = 0$ to $t = T_{MRCA}$. The right column shows inferred ancestral state probabilities from 1980 to 2020. m_Q: multiplier m for transition rates between Blantyre zones (μ_Z) relative to Blantyre-to-Global transition rates (μ_2). m lambda: multiplier for diversification rate in the Global state (λ_G) relative to the baseline diversification rate (λ).

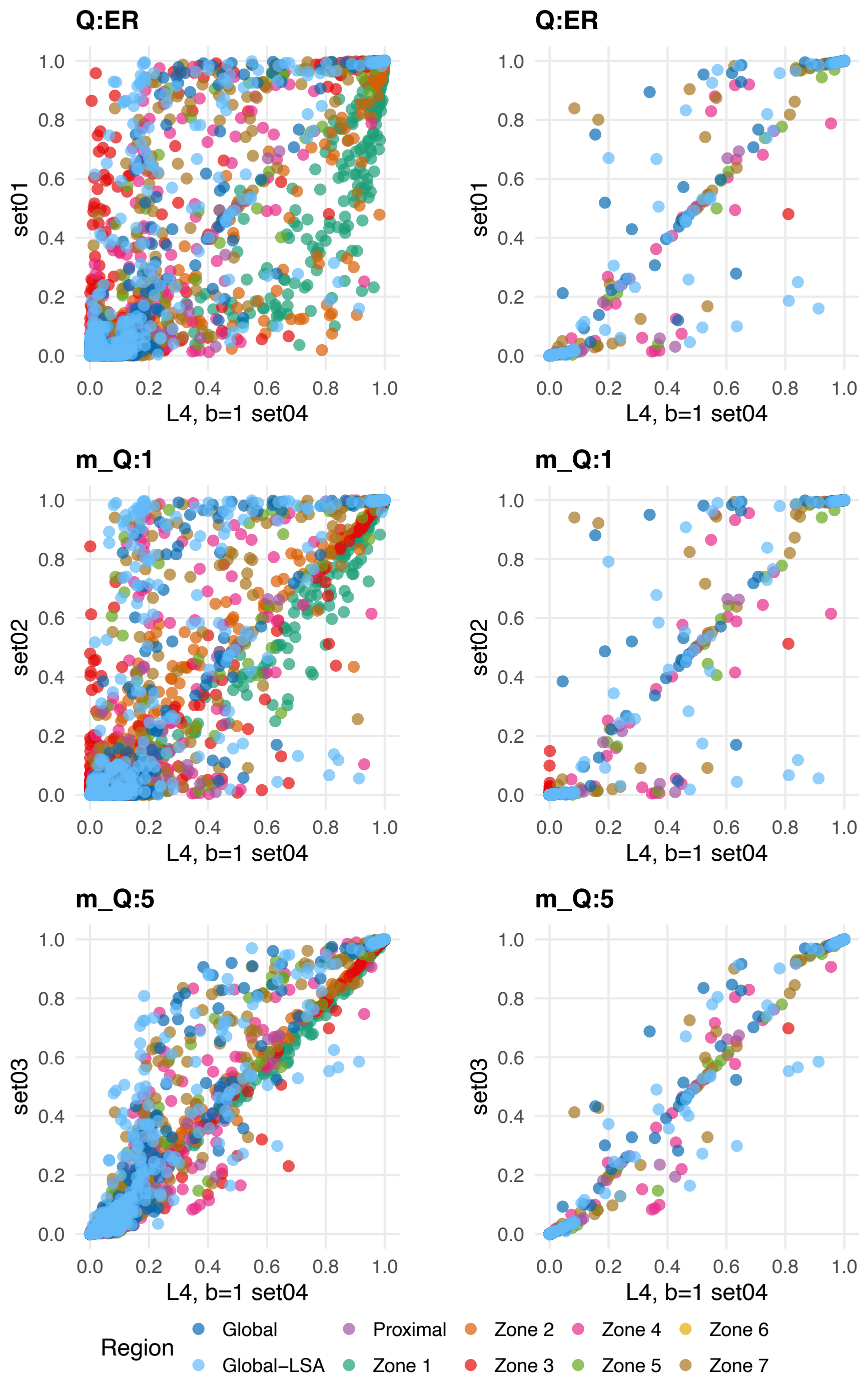

Supplemental Figure 9: Comparison between ancestral state inference results of Lineage 4, $b = 1$, parameter set 4 and modifications of Q under a diversification rate independent of state ($\lambda_G = 0.005$, see Table S3). The left column shows inferred ancestral state probabilities from $t = 0$ to $t = T_{MRCA}$. The right column shows inferred ancestral state probabilities from 1980 to 2020. Q:ER: equal rates matrix; m_Q: multiplier m for transition rates between Blantyre zones (μ_Z) relative to Blantyre–to–Global transition rates (μ_2) in the hierarchical Q model.

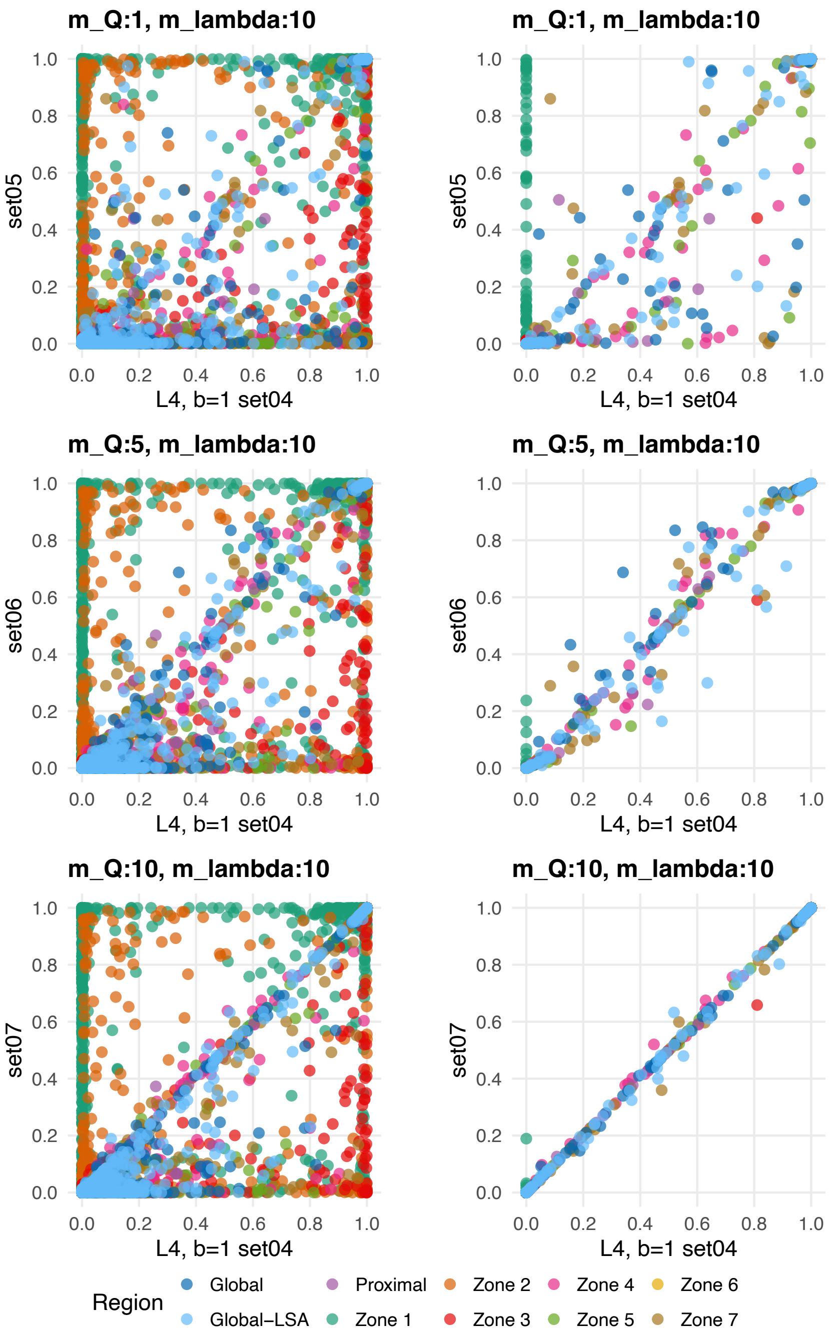

Supplemental Figure 10: Comparison between ancestral state inference results of Lineage 4, $b = 1$, parameter set 4 and modifications of Q under an elevated diversification rate for the Global state ($\lambda_G = 0.05$, see Table S3). The left column shows inferred ancestral state probabilities from $t = 0$ to $t = T_{MRCA}$. The right column shows inferred ancestral state probabilities from 1980 to 2020. m_Q: multiplier m for transition rates between Blantyre zones (μ_Z) relative to Blantyre-to-Global transition rates (μ_2). m_lambda: multiplier for diversification rate in the Global state (λ_G) relative to the baseline diversification rate (λ).

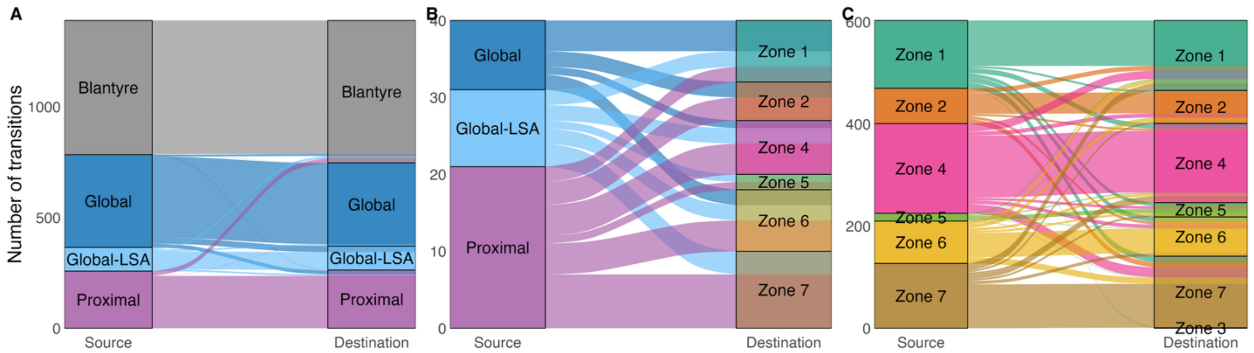

Supplemental Figure 11: Movement of Lineage 4 between geographic regions from 1999 to 2020 as inferred from SAASI with sequences from Proximal countries sampled with probability equal to their contribution to total notifications ($b = 1$). Alluvial plots are generated from the same data in Supplemental Figure 2.

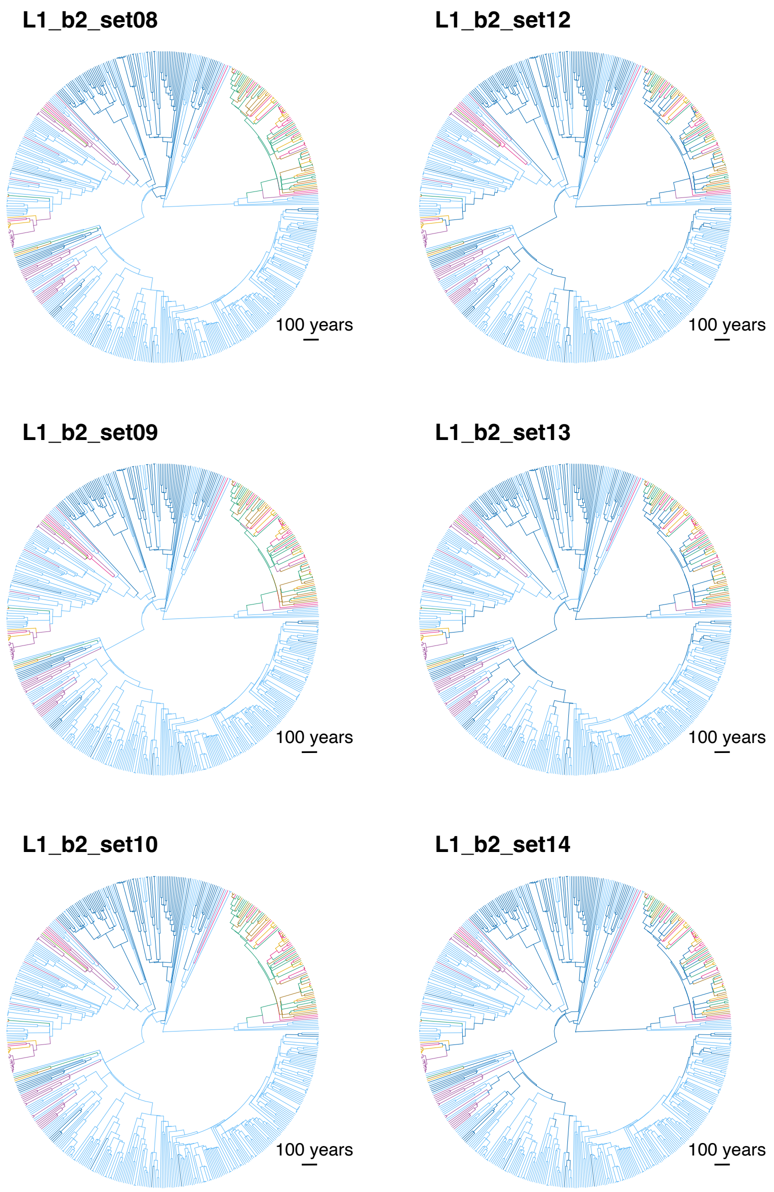

Supplemental Figure 12: SAASI trees for Lineage 1 under alternative parameterizations for the model with the same number of Proximal sequences as in the main text ($b = 2$). Parameter sets correspond to those in Table S3. In addition to an equal rates model for the stochastic rate matrix (Q) describing lineage movements (parameter set 8), parameter variations include an elevated diversification rate in the Global region (λ_G) and different multipliers for movement within Blantyre relative to the Global-to-Blantyre transitions (m). In the left column ($\lambda_G = 0.006$), the rows correspond to an equal-rates model for Q, and hierarchical structures with Blantyre multipliers of $m = 1$ and 5; the right column ($\lambda_G = 0.06$) has hierarchical models with Blantyre multipliers for $m = 1, 5,$ and 10. The analysis in the main text uses $\lambda_G = 0.006$, Blantyre multiplier $m = 10$ with a hierarchical Q matrix. Colors correspond to the same locations as in Supplemental Figure 1.

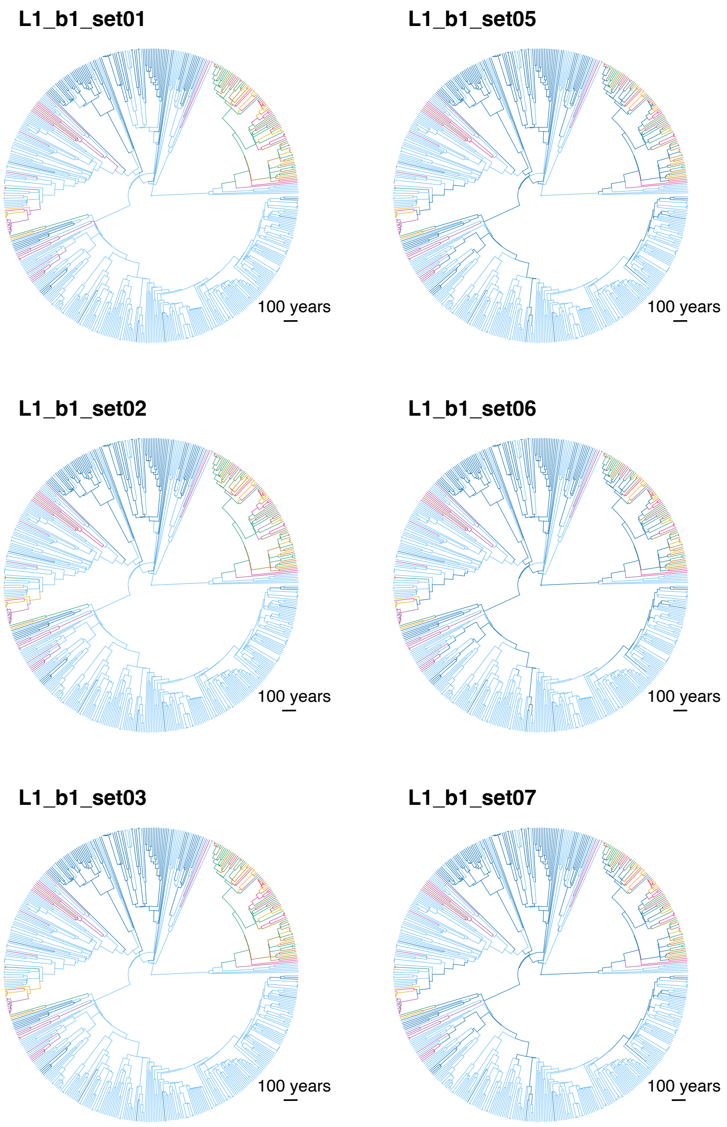

Supplemental Figure 13: SAASI trees for Lineage 1 under alternative parameterizations for the model with fewer Proximal sequences than the analysis in the main text ($b = 1$). Parameter sets correspond to those in Table S3. *In addition to an equal rates model for the stochastic rate matrix (Q) describing lineage movements (parameter set 1), parameter variations include an elevated diversification rate in the Global region (λ_G) and different multipliers for movement within Blantyre relative to the Global-to-Blantyre transitions (m). In the left column ($\lambda_G = 0.004$), the rows correspond to an equal-rates model for Q, and hierarchical structures with Blantyre multipliers of $m = 1$ and 5; the right column ($\lambda_G = 0.04$) has hierarchical models with Blantyre multipliers for $m = 1, 5,$ and 10. Colors correspond to the same locations as in Supplemental Figure 1.*

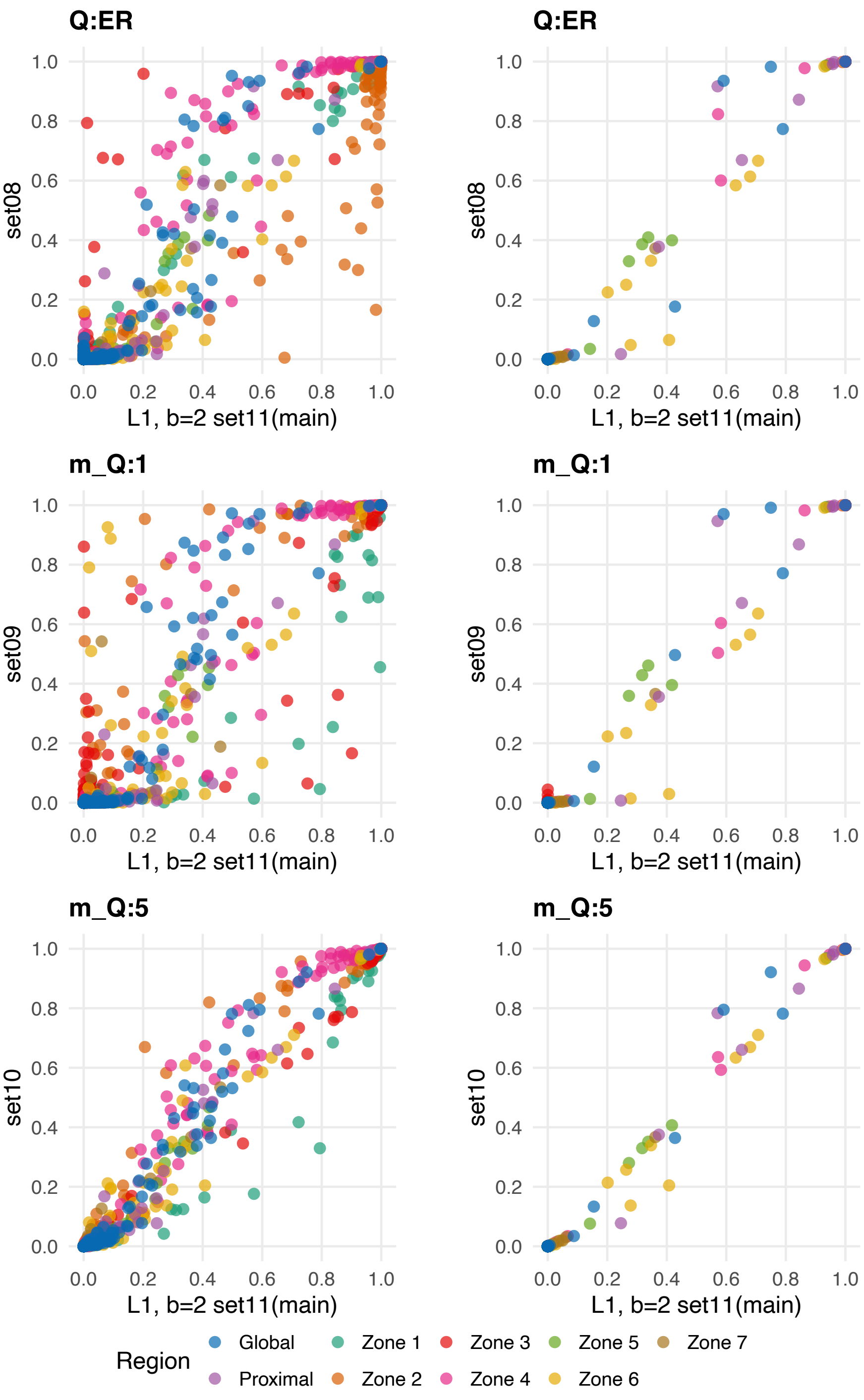

Supplemental Figure 14: Comparison between ancestral state inference results of Lineage 1, $b = 2$, parameter set 11 (main analysis) and modifications of Q under a diversification rate independent of state ($\lambda_G = 0.006$, see Table S3). The left column shows inferred ancestral state probabilities from $t = 0$ to $t = T_{MRCA}$ for the main settings and other settings. The right column shows inferred ancestral state probabilities from 1980 to 2020. Q:ER: equal rates matrix; m_Q: multiplier m for transition rates between Blantyre zones (μ_Z) relative to Blantyre–to–Global transition rates (μ_2) in the hierarchical Q model.

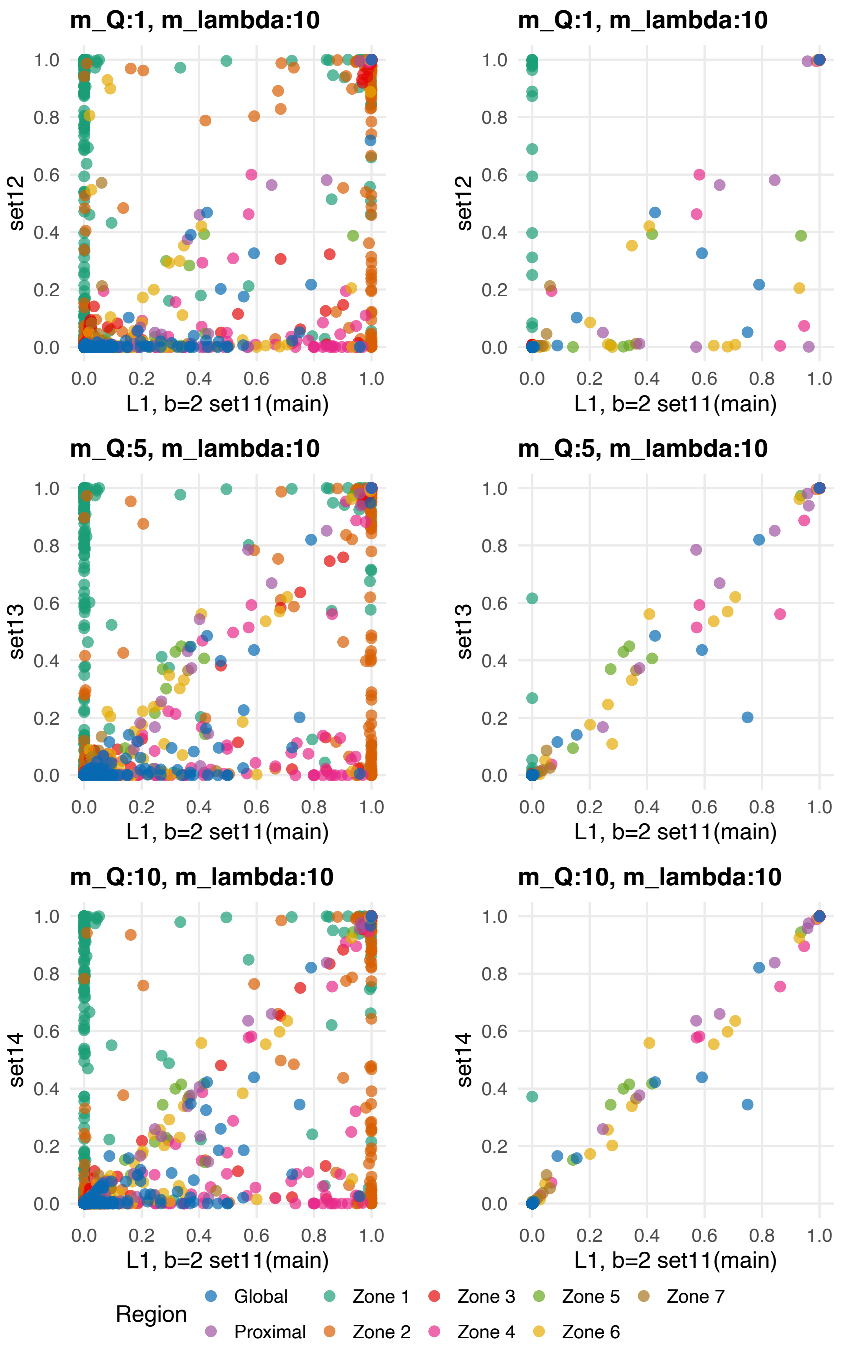

1004

1005 *Supplemental Figure 15: Comparison between ancestral state inference results of Lineage*
1006 *1, $b = 2$ parameter set 11 (main analysis) and modifications of Q under an elevated*
1007 *diversification rate for the Global state ($\lambda_G = 0.06$, see Table S3). The left column shows*
1008 *inferred ancestral state probabilities from $t = 0$ to $t = T_{MRCA}$ for the main settings and other*
1009 *settings. The right column shows inferred ancestral state probabilities from 1980 to 2020.*
1010 *m_Q: multiplier m for transition rates between Blantyre zones (μ_Z) relative to Blantyre-to-*
1011 *Global transition rates (μ_2). m_lambda: multiplier for diversification rate in the Global state*
1012 *(λ_G) relative to the baseline diversification rate (λ).*

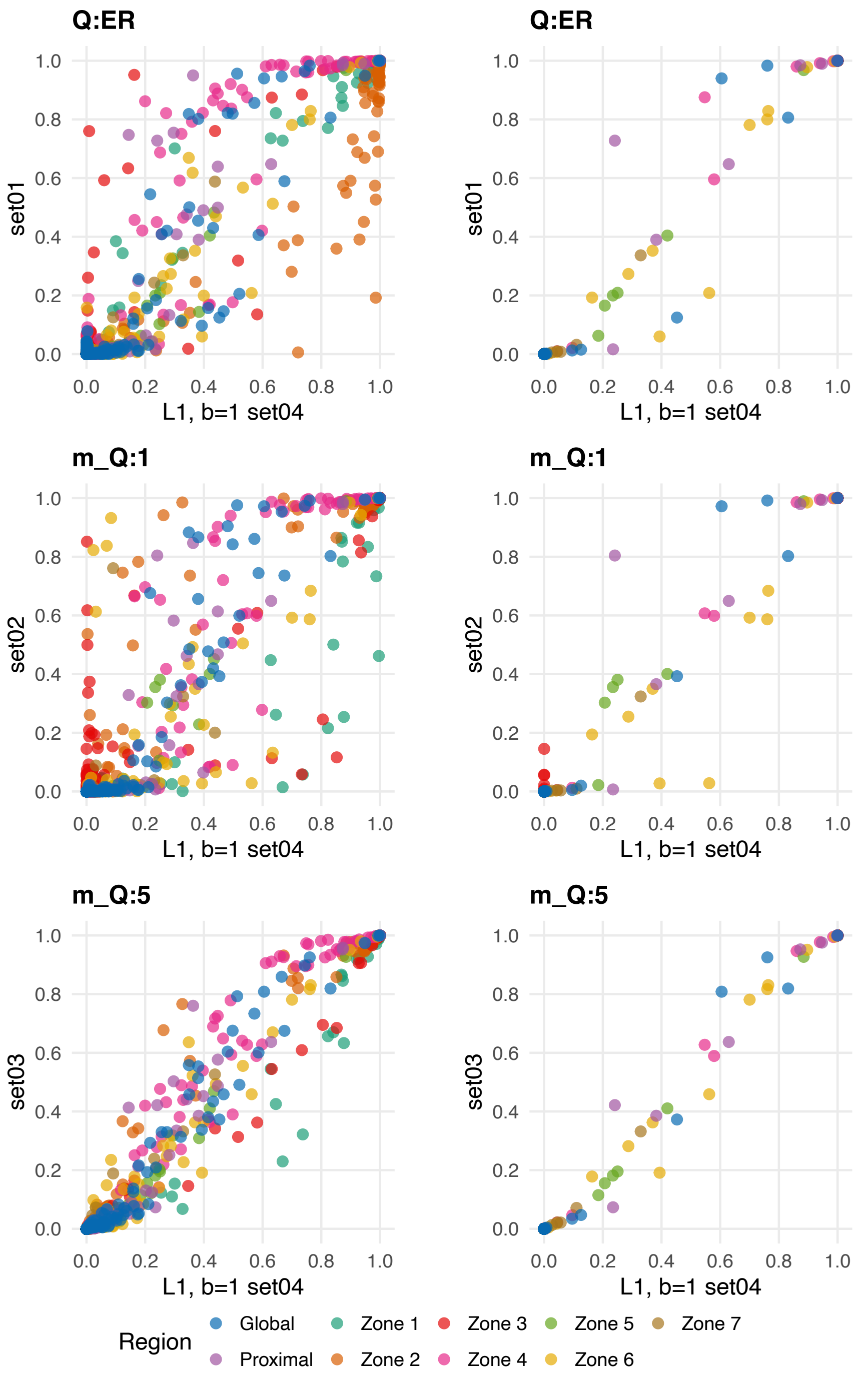

Supplemental Figure 16: Comparison between ancestral state inference results of Lineage 1, $b = 1$, parameter set 4 and modifications of Q under a diversification rate independent of state ($\lambda_G = 0.004$, see Table S3). The left column shows inferred ancestral state probabilities from $t = 0$ to $t = T_{MRCA}$ for the main settings and other settings. The right column shows inferred ancestral state probabilities from 1980 to 2020. Q:ER: equal rates matrix; m_Q: multiplier m for transition rates between Blantyre zones (μ_Z) relative to Blantyre–to–Global transition rates (μ_2) in the hierarchical Q model.

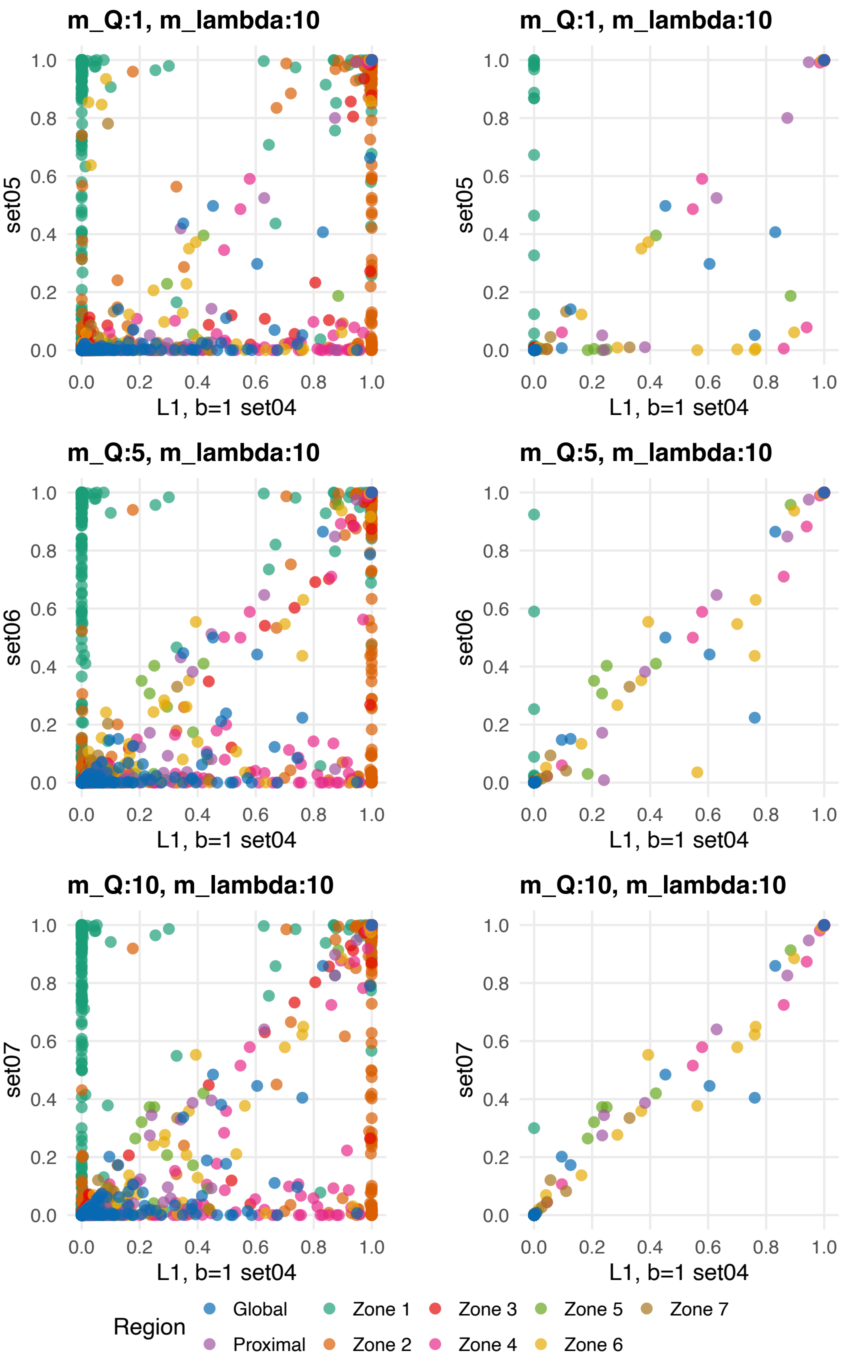

Supplemental Figure 17: Comparison between ancestral state inference results of Lineage 1, $b = 1$, parameter set 4 and modifications of Q under an elevated diversification rate for the Global state ($\lambda_G = 0.04$, see Table S3). The left column shows inferred ancestral state probabilities from $t = 0$ to $t = T_{MRCA}$ for the main settings and other settings. The right column shows inferred ancestral state probabilities from 1980 to 2020. m_Q: multiplier m for transition rates between Blantyre zones (μ_Z) relative to Blantyre-to-Global transition rates (μ_2). m lambda: multiplier for diversification rate in the Global state (λ_G) relative to the baseline diversification rate (λ).

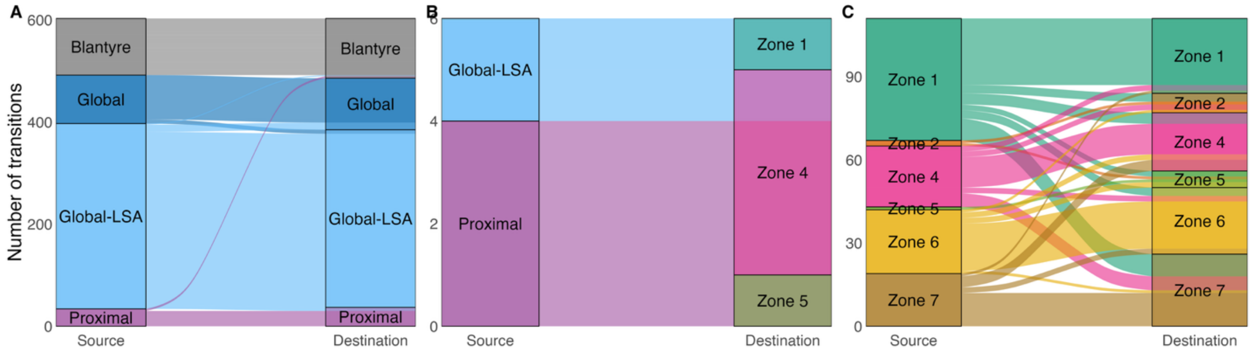

Supplemental Figure 18: Alluvial plots summarizing movements of Lineage 1 between geographic regions from 1999 to 2020 with sequences from Proximal countries sampled with probability equal to their contribution to total notifications ($b = 1$). Plots are generated from the same data in Supplemental Figure 2.

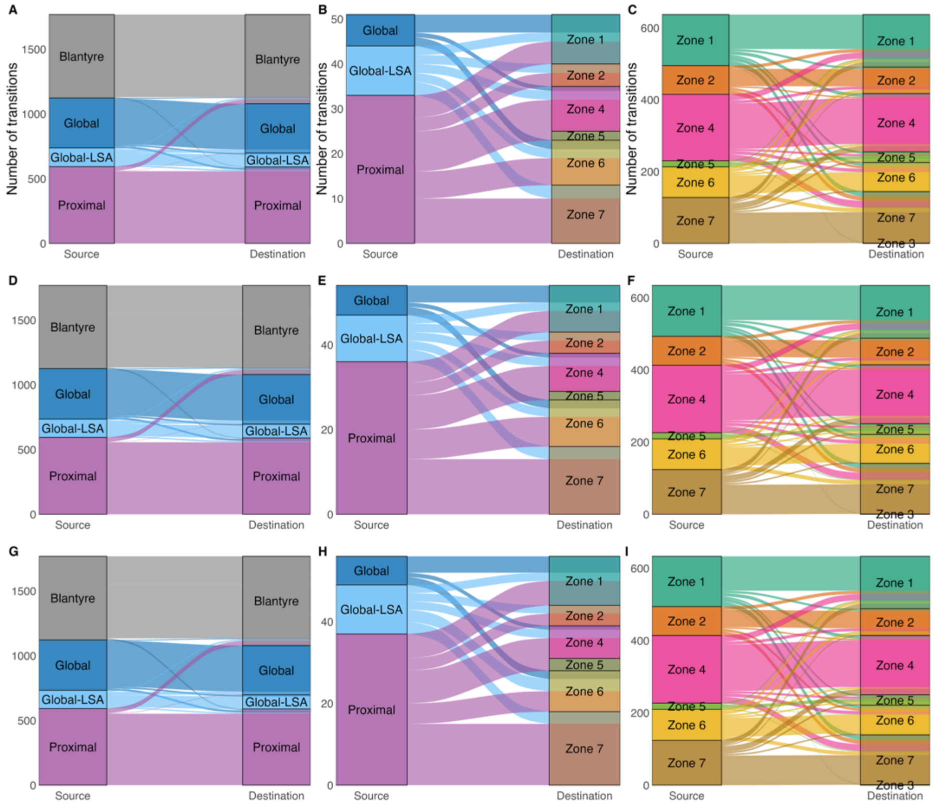

Supplemental Figure 19: Estimated lineage movements are insensitive to increases in the transition rate from the proximal region to the Blantyre zones (the $Q_{1,8}$ entry of the stochastic rate matrix, which equals μ_1 in the main analysis). The top row (A,B,C) is the same analysis as in Figure 6, the middle row (D,E,F) is calculated using a transition rate that is twice as large as the estimated value in the main text, and in the bottom row (G,H,I) the rate is three times higher.

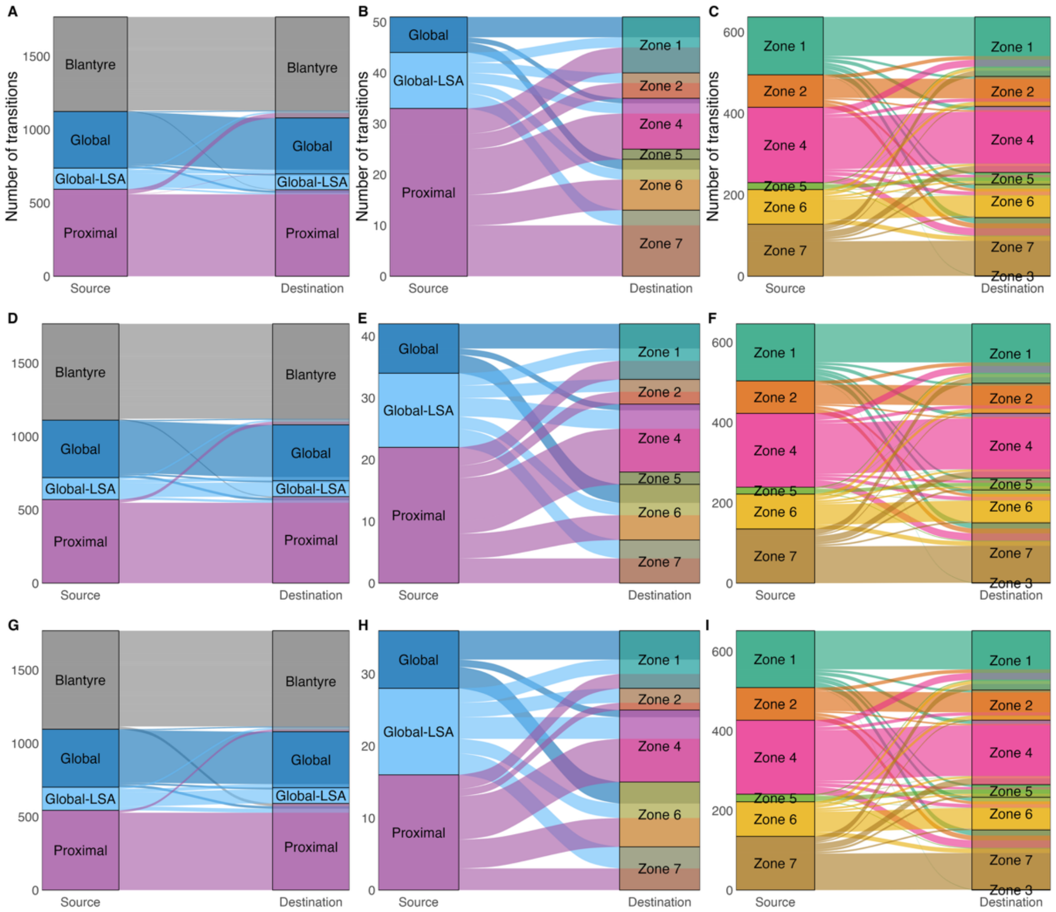

Supplemental Figure 20: The rate of lineage movement from Blantyre to the proximal region (the $Q_{8,1}$ entry of the stochastic rate matrix, which equals μ_1 in the main analysis) affects the number of importations into Blantyre, but not the transmission dynamics between the zones within Blantyre. The top row (A,B,C) is the same analysis as in Figure 6, the middle row (D,E,F) uses a rate that is twice the estimated value, and in the bottom row (G,H,I) that rate is three times higher.

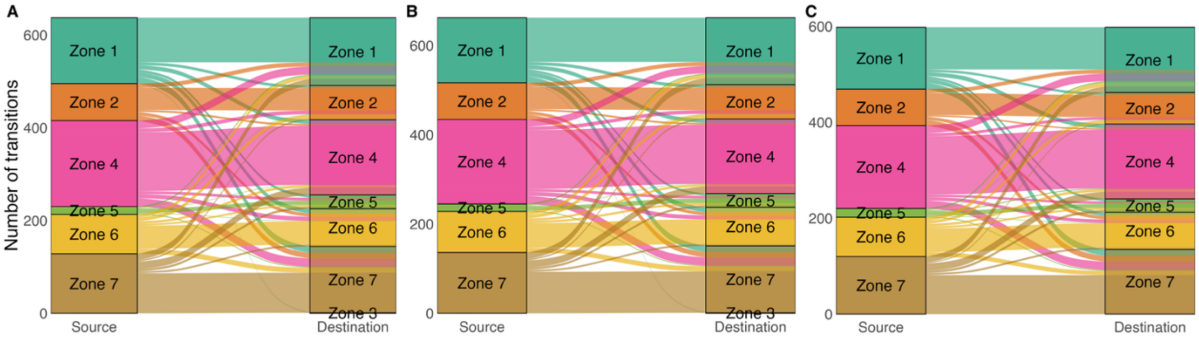

Supplemental Figure 21: Lineage movements between zones in Blantyre are insensitive to large changes in the transition rates between the proximal region and Blantyre (the $Q_{1,8}$ and the $Q_{8,1}$ entries of the stochastic rate matrix, which equal μ_1 in the main analysis). Panel A

corresponds to the analysis displayed in Figure 6, panel B uses a transition rate from the Blantyre zones to the proximal region ($Q_{8,1}$) that is ten times the estimated value, and panel C uses a transition rate from the proximal region into the zones ($Q_{1,8}$) that is 10 times the estimated value.

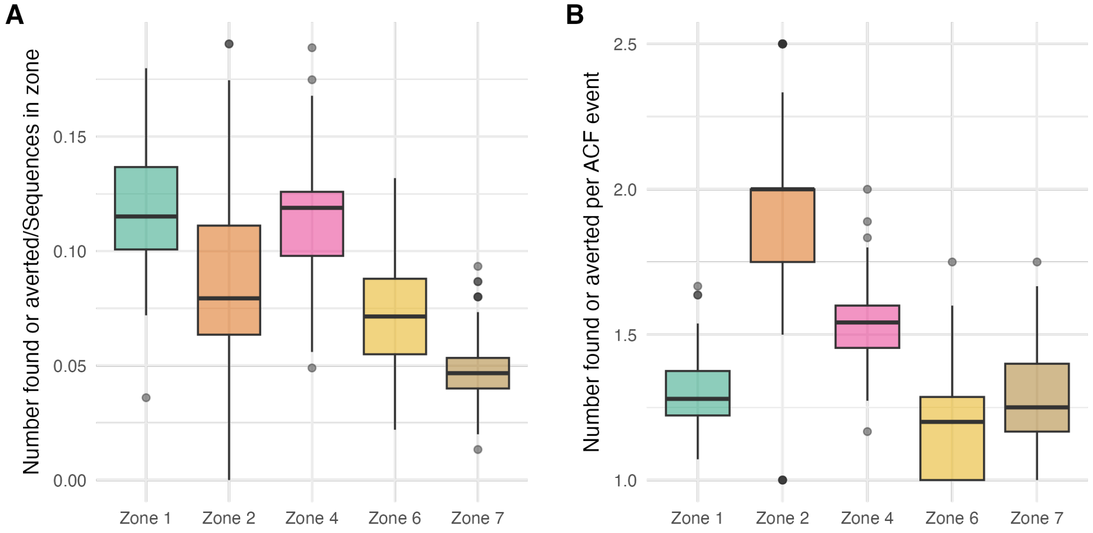

Supplemental Figure 22: A simulated zone-specific ACF intervention from 2011 through the end of 2014 based on analysis using fewer sequences from Proximal countries ($b = 1$). The impact of ACF is quantified according to number of cases averted in a particular zone relative to the number of sequences from that zone in the data (A), and in terms of the number of infections identified or prevented relative to the number of infections identified through ACF (B). In (B), Number found or averted includes individuals directly identified through the ACF intervention. Zones 3 and 5 are omitted due to small numbers of sequences from those zones in the data. Interventions are simulated using the same data in Supplemental Figure 3. The probability of successfully identifying and treating active lineages is set to 40%. See Methods for a description of the intervention simulation.

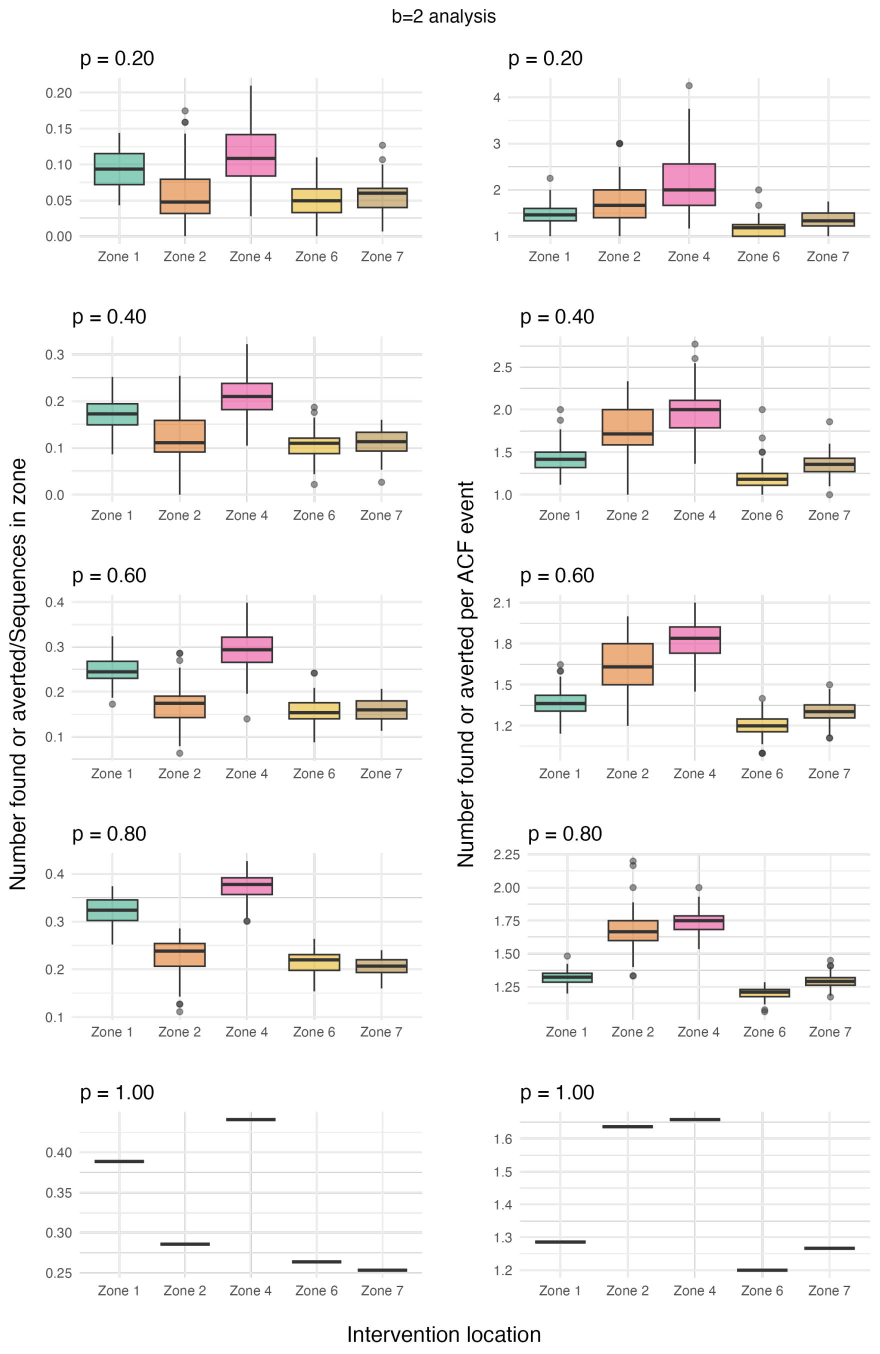

Supplemental Figure 23: Simulated zone-specific ACF interventions for different values of p, the probability of successfully identifying and treating cases before onward transmission, using the SAASI analysis from the main text ($b = 2$). The p=0.60 simulations correspond to Figure 8 of the main text. The impacts of ACF are quantified in the same manner as in Figure 8 and Supplemental Figure 22. Interventions are simulated using the same data in Figure 4 and Figure 5. See Methods for a description of the intervention simulation.

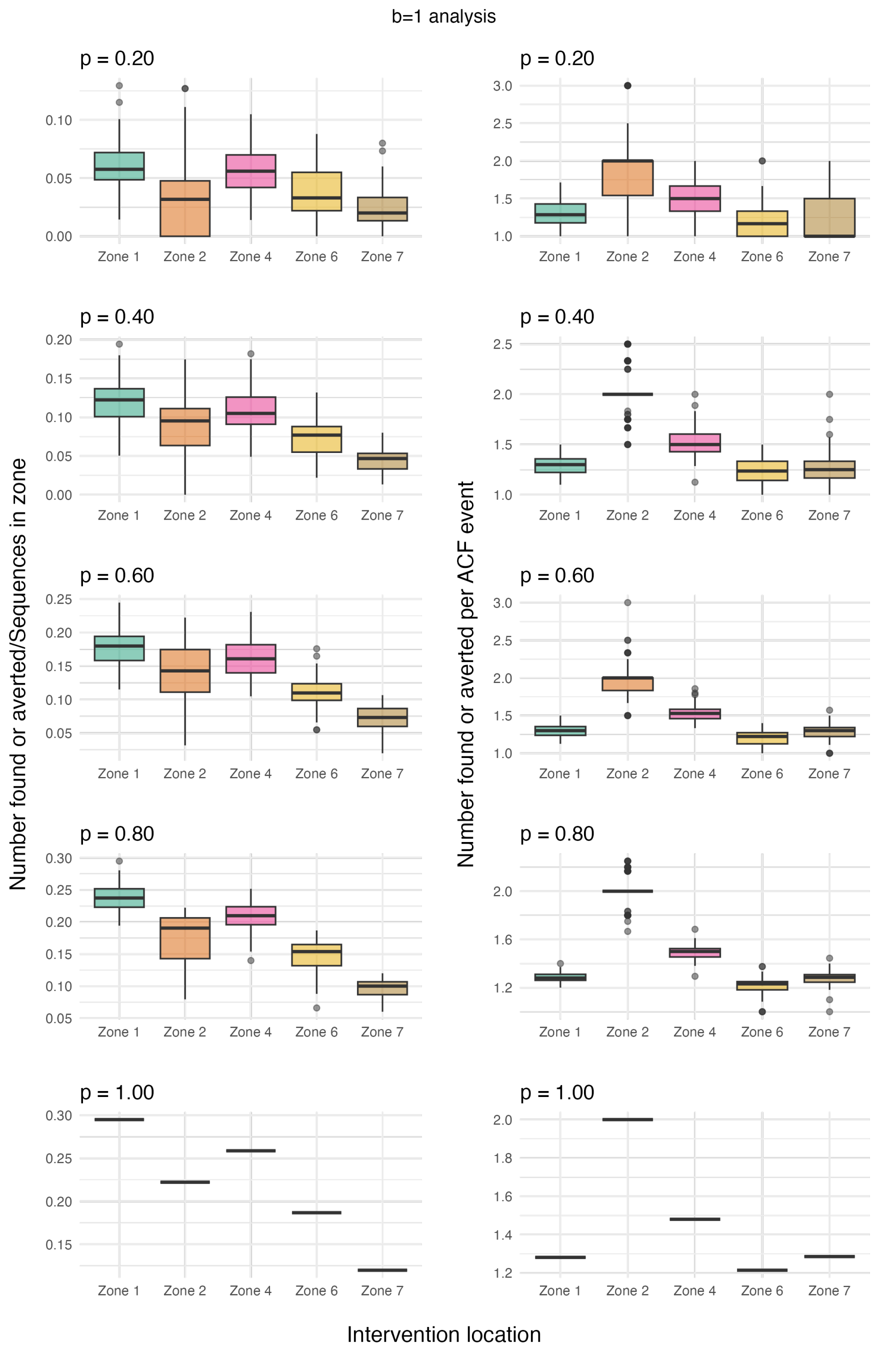

Supplemental Figure 24: Simulated zone-specific ACF interventions for different values of p, the probability of successfully identifying and treating cases before onward transmission, using the SAASI analysis with fewer sequences from Proximal countries ($b = 1$). The p=0.40 simulations correspond to Supplemental Figure 22. The impacts of ACF are quantified in the same manner as in Figure 8 and Supplemental Figure 22. Zones 3 and 5 are omitted due to small numbers of sequences from those zones in the data. Interventions are simulated using the same data in Supplemental Figure 3. See Methods for a description of the intervention simulation.

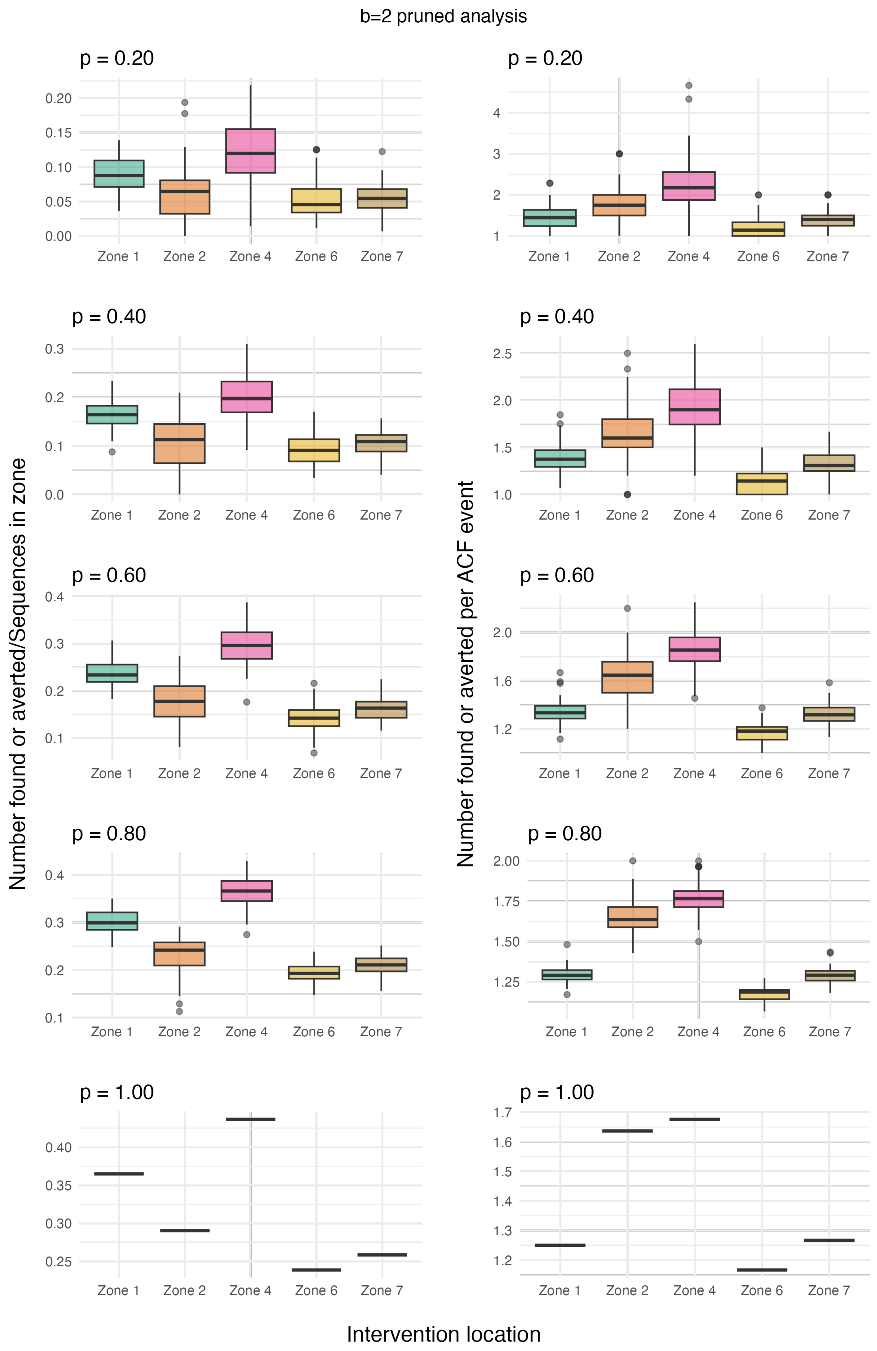

Supplemental Figure 25: Simulated zone-specific ACF interventions for different values of p using the SAASI analysis from the main text ($b = 2$) after removing thirteen sequences with ambiguous zone information in Blantyre. The impacts of ACF are quantified in the same manner as in Figure 8 and Supplemental Figure 22. Interventions are simulated using the same data in Figure 4 and Figure 5 after pruning away sequences with ambiguous zone information. See Methods for a description of the intervention simulation.

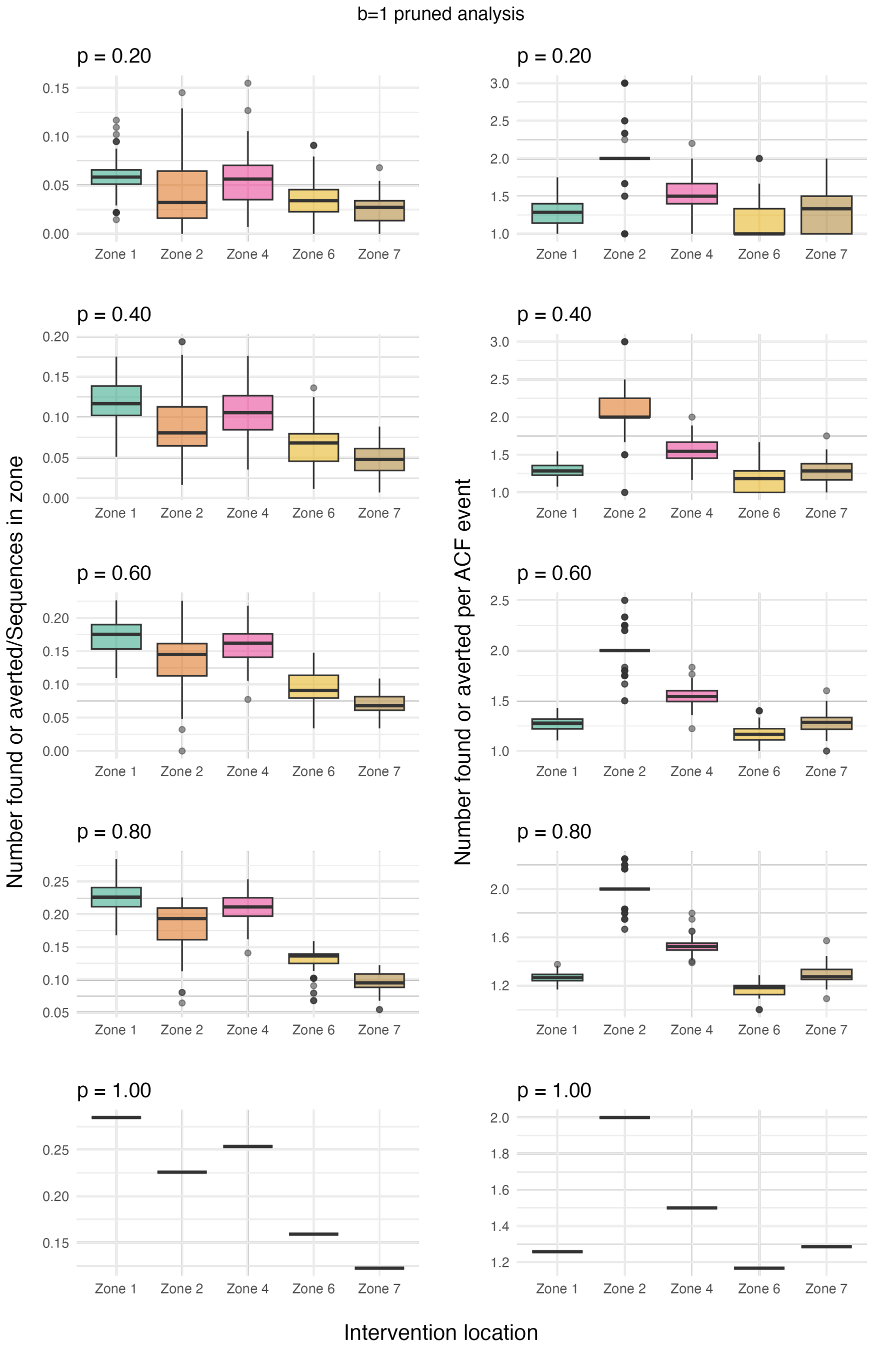

Supplemental Figure 26: Simulated zone-specific ACF interventions for different values of p using the SAASI analysis with fewer sequences from Proximal countries ($b = 1$) and after removing thirteen sequences with ambiguous zone information in Blantyre. The impacts of ACF are quantified in the same manner as in Figure 8 and Supplemental Figure 22. Zones 3 and 5 are omitted due to small numbers of sequences from those zones in the data. Interventions are simulated using the same data in Supplemental Figure 3 after pruning away sequences with ambiguous zone information. See Methods for a description of the intervention simulation.
